# Human brain activity reflecting facial attractiveness from skin reflection

**DOI:** 10.1038/s41598-021-82601-w

**Published:** 2021-02-22

**Authors:** Yuichi Sakano, Atsushi Wada, Hanako Ikeda, Yuriko Saheki, Keiko Tagai, Hiroshi Ando

**Affiliations:** 1 Center for Information and Neural Networks (CiNet), National Institute of Information and Communications Technology, and Osaka University, Suita, Osaka Japan; 2 Graduate School of Frontier Biosciences, Osaka University, Suita, Osaka Japan; 3 Advanced Research Center, Shiseido Global Innovation Center, Yokohama, Kanagawa Japan

**Keywords:** Perception, Reward, Colour vision, Human behaviour, Skin manifestations

## Abstract

Facial attraction has a great influence on our daily social interactions. Previous studies have mainly focused on the attraction from facial shape and expression. We recently found that faces with radiant skin appear to be more attractive than those with oily-shiny or matte skin. In the present study, we conducted functional magnetic resonance imaging (fMRI) and psychological experiments to determine the human brain activity that reflects facial attractiveness modulated by these skin reflection types. In the fMRI experiment, female subjects were shown successive images of unfamiliar female faces with matte, oily-shiny, or radiant skin. The subjects compared each face with the immediately preceding face in terms of attractiveness, age, and skin reflection, all based on the skin. The medial part of the orbitofrontal cortex (mOFC) was significantly more active when comparing attractiveness than when comparing skin reflection, suggesting that the mOFC is involved in processing facial attractiveness from skin reflection. In the psychological experiment, attractiveness rating was highest for radiant skin, followed by oily-shiny, and then matte skin. Comparison of the results of these experiments showed that mOFC activation level increased with attractiveness rating. These results suggest that the activation level of the mOFC reflects facial attractiveness from skin reflection.

## Introduction

Facial attraction has a great influence on a wide range of our daily social interactions^1,2^_,_ including mate choices^3^ and more general decisions about other types of social partners^2^ such as hiring^4,5^.

How do we judge facial attractiveness? Previous studies have mainly focused on the effects of global facial shape (e.g. averageness^6–13^, symmetry^7,9^, and sexually dimorphic shape cues^14^) and expression^15,16^ while some others have examined the effects of facial skin colour^17–27^ (for review, see Thornhill and Gangestad, 1999^1^ and Little et al., 2011^2^).

In the present study, we focused on the effects of skin reflection on facial attractiveness. Given that facial attractiveness reflects the degree of health^1,2,28^, facial skin reflection could affect facial attractiveness because it gives some hints about the health conditions of an individual. That is, highly diffuse reflection indicates not only a low melanin content but also a large amount of moisture in the skin and fine skin texture^29,30^, whilst specular reflection implies the sebum distributed on the skin surface^31^. A substantial amount of moisture in the skin reflects both soundness of water flux regulation and a high capability for water retention^32–34^. Such flux regulation and water retention contribute to protection against desiccation and pathogen challenges^32,35^. Similarly, sebum, when mixed with sweat, becomes a thin acidic film (“acid mantle”) on the skin that acts as a barrier to pathogenic microbes^36–41^. In addition, sebum plays an important role in keeping the body temperature constant (“thermoregulation”^42^). Therefore, it is reasonable to expect that skin reflection, a visual cue to health, may affect facial attractiveness.

**Figure 1:**
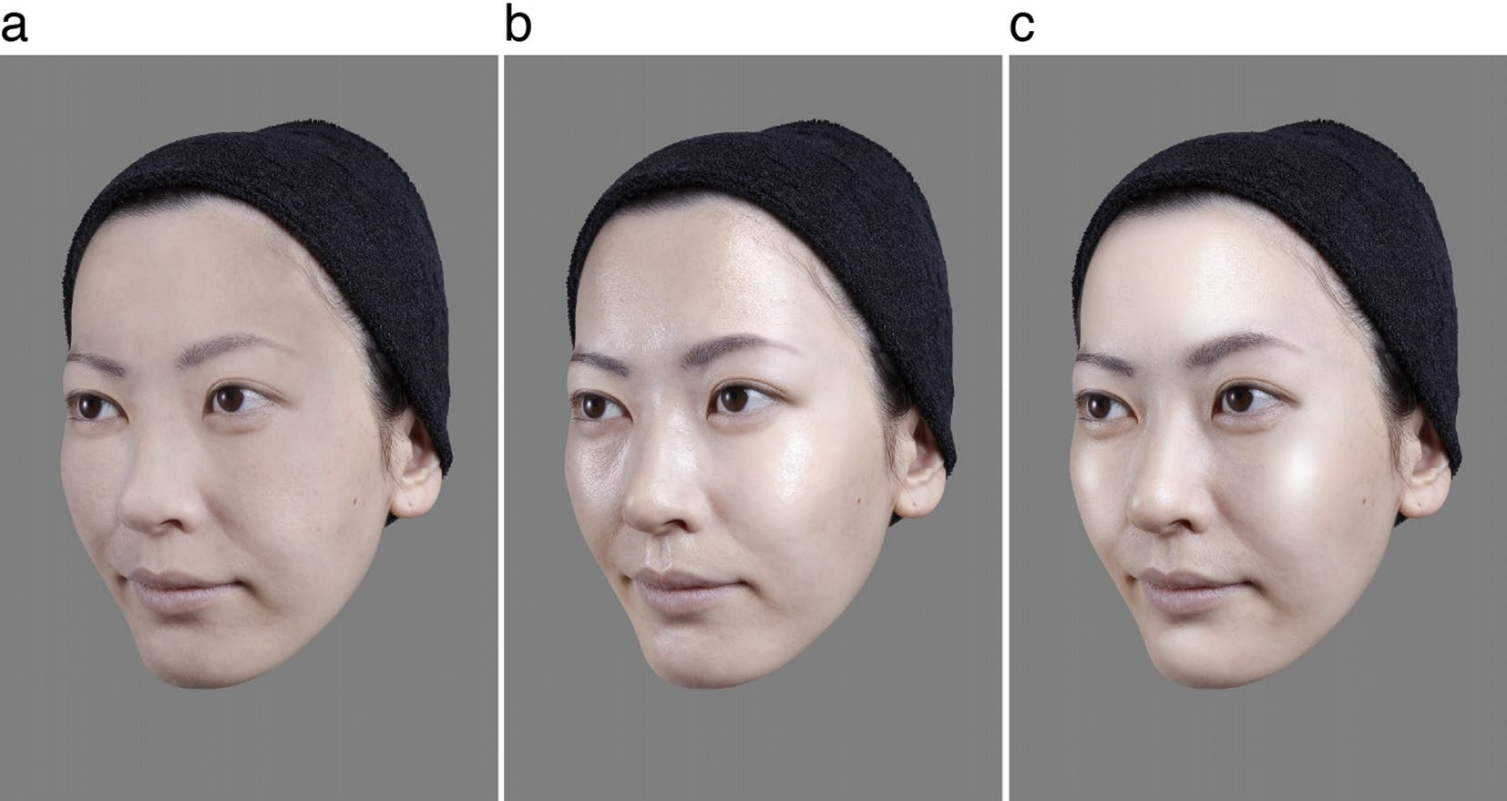
Examples of stimuli used in both fMRI and psychological experiments. The facial skin was matte **(a)**, oily-shiny **(b)**, or radiant **(c)**.

Indeed, we recently found that skin reflection substantially affects facial attractiveness for female observers^43^. That study, which employed a total of 160 female subjects in their 30s to 40s along with a pairwise comparison method showed that attractiveness increases in the order of faces with matte, oily-shiny, and then radiant skin (Fig. 1). These types of reflection are unique to skin^30,44–50^, which has complex internal and external structures^29,30,51^. Such categorization or concepts of skin reflection are becoming pervasive, typically among women in many countries, and are inherently determined by visual impression rather than physical reflectance parameters^30,44–50^. Matte skin makes little impression of gloss. Oily-shiny skin makes an impression of substantial gloss and sebum without a sense of unity of skin and cosmetic foundation^49^ (if worn). It is often mentioned that people with radiant skin appear to have an internal glow^48^. Physically, oily-shiny skin tends to show high specular and low diffuse reflectances while radiant skin tends to exhibit high specular and high diffuse reflectances^30^.

However, both the neural basis and the brain activity associated with the effects of skin reflection on facial attractiveness remain unknown. Previous brain studies on facial attraction from other or unspecified factors have explored the human brain for the neural basis of processing facial attractiveness. Many studies have reported increases in functional magnetic resonance imaging (fMRI) or positron emission tomography (PET) signal when viewing attractive compared to unattractive faces, or when judging facial attractiveness compared with other features; these are localized within brain regions in reward systems such as the orbitofrontal cortex (OFC) ^52–62^, the nucleus accumbens^53,59^ in the ventral striatum^16^, the caudate nucleus^62^, the pallidum^16^, the thalamus^16,55,56^, the anterior^57,60,61^ and posterior cingulate cortex^54^, the anterior insular cortex^52,57,63^, and the medial prefrontal cortex^54,57,58,63^_._ Other studies have identified activation within other brain regions in the “face network” ^64–68^ such as the middle temporal gyrus^69^ and the fusiform gyrus^63,70,71^. For example, it has been reported that fMRI activation level in the medial part of the orbitofrontal cortex (mOFC) was not only higher in the high attractiveness conditions than in the low attractiveness conditions, but also enhanced when viewing a smiling facial expression^54^. Another fMRI study^63^ reported that whilst activity in many cortical regions was correlated parametrically with the degree of facial attractiveness during explicit judgement of facial beauty, ventral occipital regions remained responsive to facial beauty during judgement of facial identity. Based on such results, the authors proposed that the ventral occipital regions, including the fusiform face area (FFA) and the lateral occipital cortex (LOC), may serve as a neural trigger for effects of attractiveness in social interactions. Several studies^28,72–75^ using event-related potentials (ERPs) have also examined the effects of facial attractiveness, and it has been suggested that seemingly in the FFA and/or the occipital face area (OFA), fewer neural resources are engaged for faces with high attractiveness and averaged faces than faces with low attractiveness^73^.

In the present study, we tried to determine human brain activity that is associated with facial attractiveness modulated by the different types of skin reflection discussed previously (specifically matte, oily-shiny, and radiant skin). To achieve this goal, we carried out two experiments, as described below.

The first aim was to identify the human brain regions involved in processing facial attractiveness based on skin reflection. To do so, we conducted an fMRI experiment with an attention-based technique that utilizes the fact that paying attention to a certain feature enhances neural activation in the brain regions involved in processing that particular feature^76–82^. Therefore, when a subject conducts a task, the brain regions involved in processing a feature that is crucial to perform the task are expected to be activated because of the selective attention paid to that feature. We expected that certain regions among those that have been reported to be involved in processing facial attractiveness mentioned above may be involved in processing facial attractiveness that arises from skin reflection.

The second aim was to determine whether the activation levels of the regions identified as those that are involved in processing facial attractiveness based on skin reflection reflect the magnitude of facial attractiveness based on skin reflection. To this end, we conducted a psychological experiment in which the same subjects who participated in the fMRI experiment this time rated the absolute (rather than relative) attractiveness of all the facial images presented in the fMRI experiment. We expected that the activation levels of those regions would increase with the rated attractiveness as is the case with facial attractiveness from other factors^53,54,56–63,69^.

We also tried to determine the brain activity that reflects how the perceived age is modulated by skin reflection, since perceived age also impacts social behaviours^83–85^ and is affected by skin reflection^43,86,87^.

## Results

### Attractiveness

The first aim was to identify the human brain regions involved in processing facial attractiveness based on skin reflection. To this end, we conducted an fMRI experiment. Female subjects were shown successive unfamiliar female face images (Supplementary Fig. S1 online) with radiant, oily-shiny, or matte skin (Fig. 1). The subjects were asked to judge whether each face was higher or lower than the immediately preceding face in terms of attractiveness, age, or skin reflection, all based on skin impression (for detail on reflection, see Supplementary Fig. S2 online). The reasons for judgments being based solely on skin impression was that we aimed at identifying specific regions involved in processing facial attractiveness solely from skin reflection, rather than from any available cue, including facial global shape^62^. The different tasks (i.e., attractiveness, age, and skin reflection) were performed in different experimental blocks. In all these different task conditions, an identical set of faces was presented. Thus, we theorized that differences in the fMRI activity of a given brain region between different task conditions would not be due to different stimuli but due to differences in the feature the subject paid attention to during the tasks.

We compared the fMRI activation levels in the attractiveness task blocks with those in the skin reflection task blocks. If certain regions are more active during the attractiveness blocks than the skin reflection blocks, this suggests that these regions are involved in processing facial attractiveness based on skin reflection. We subtracted the activation in the skin reflection blocks from the activation in the attractiveness blocks to exclude the possible activation in the regions involved in estimation of skin reflection, which should have preceded the processing of attractiveness itself.

**Figure 2:**
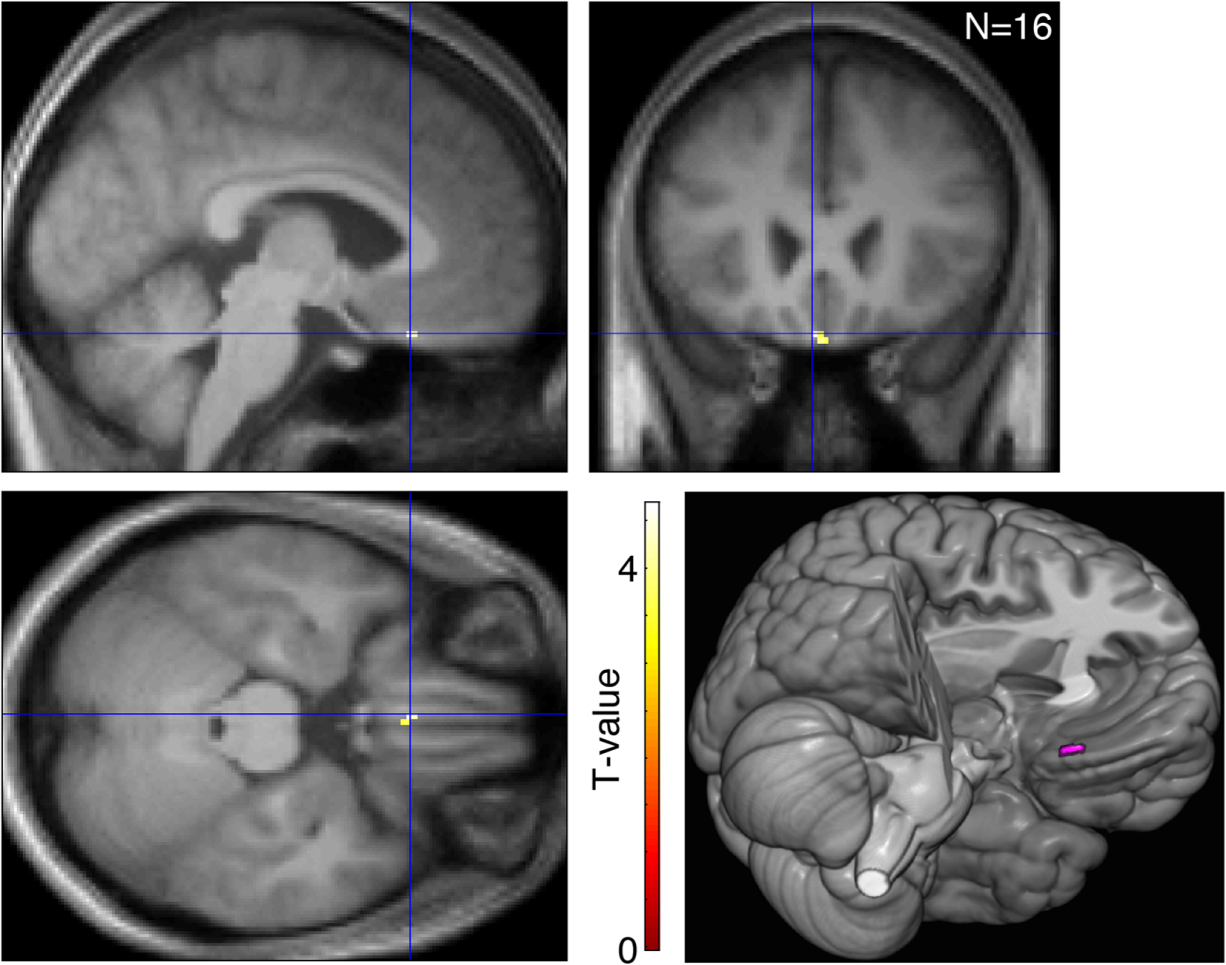
The mOFC was the only region that was significantly more activated during the attractiveness task block than during the reflection task block. The crosshairs indicate the peak voxel of the cluster (MNI: − 4, 24, − 24). An effect size metric Hedges' *g*_*av*_^139^ of this contrast in the beta values in this voxel was 1.138. The lower-right panel image was rendered using MRIcroGL software (https://www.mccauslandcenter.sc.edu/mricrogl/home).

As shown in Fig. 2, the human frontal cortex, specifically the mOFC was significantly more active when judging attractiveness than when judging skin reflection (MNI − 4, 24, − 24; cluster of 16 voxels; *t*_15_ = 4.66; Hedges' *g*_*av*_ = 0.844; Brodmann area 11; left gyrus rectus according to AAL atlas^88^).

**Figure 3:**
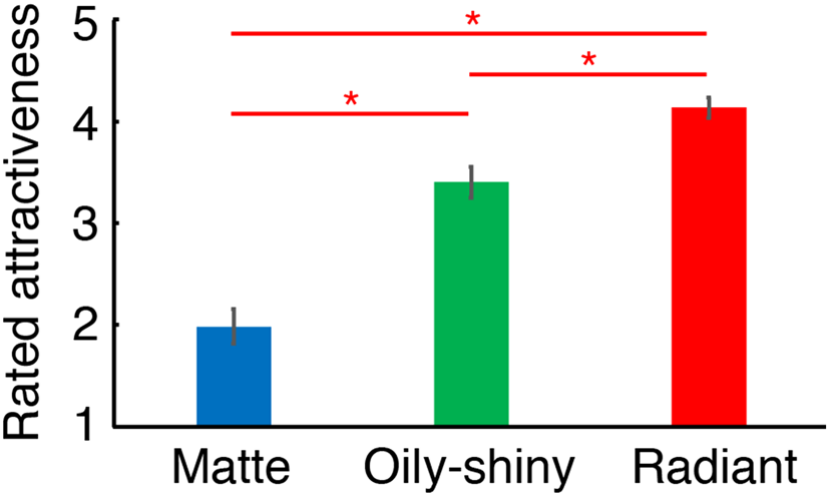
Rated attractiveness of faces with matte, oily-shiny, and radiant skin. Error bars indicate ± 1 SEM across subjects. *p < 0.05. Values of the effect size measures *η*_G_^2^ and *ω*_G_^2^ of all skin reflection types were 0.647 and 0.631, respectively^140,141^. Hedges' *g*_*av*_^139^ was 2.088, 1.348, and 3.671, for oily-shiny vs. matte, radiant vs. oily-shiny, and radiant vs. matte, respectively.

Our second aim was to examine whether the activation level of the mOFC reflects the magnitude of facial attractiveness from skin reflection. To this end, we conducted a psychological experiment in which the same subjects who participated in the fMRI experiment rated the absolute (rather than relative) attractiveness of all the facial images presented in the fMRI experiment. The rated attractiveness of faces was highest with radiant, then oily-shiny, then matte skin (Fig. 3; two-way repeated-measures ANOVA with independent variables of skin reflection and facial model, *F*(2,30) = 568.46, *p* < 0.0001; Tukey's HSD post-hoc test for skin reflection, all *p* < 0.05). For results of other tasks including perceived age and perceived skin reflection, results of ANOVAs for data from all the rating tasks, and relationships between the rating results of different tasks, see Supplementary Fig. S3, Table S1, and Fig. S4 online, respectively.

**Figure 4:**
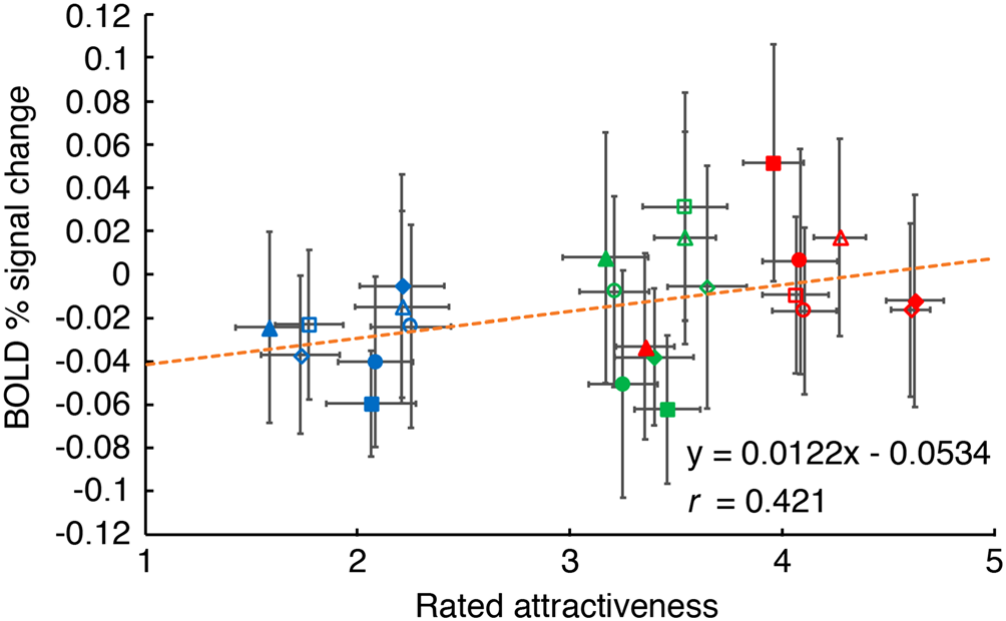
Relationship between rated attractiveness and activation in mOFC. Error bars indicate ± 1 SEM across subjects.

To examine whether the level of activity in the mOFC reflects the magnitude of facial attractiveness based on skin reflection, we compared the mOFC activation for all 24 facial images with the attractiveness rating from the psychological experiment. Activity in the mOFC was positively correlated with attractiveness (one-tailed correlation test, *r* = 0.421, *p* = 0.020, Fig. 4). This finding was also confirmed using a simple linear regression analysis with a one-tailed slope test (*p* = 0.020).

We also compared the mOFC activation between the three skin reflection types. Although the mOFC activation appeared to increase in the order of faces with matte, oily-shiny, and then radiant skin as the attractiveness rating did (compare Supplementary Fig. S5 online with Fig. 3), the differences between the different skin reflection types were not statistically significant due to relatively large individual differences (two-way repeated-measures ANOVA, *F*(2, 30) = 1.203, *p* = 0.302, Supplementary Fig. S5 online).

### Age

Similarly, to identify regions involved in judging facial age from skin reflection, we contrasted the activation levels in the age blocks with those in the skin reflection blocks. This contrast induced significant activity in regions that have been reported to be involved in processing facial age from unspecified cues from in a movie of one face morphing into another^89^ (Supplementary Fig. S6 online, Supplementary Table S2 online). However, this contrast also induced activity in many other regions that have been reported to be involved in orienting attention and executive control of attention for general purposes, rather than specifically for age processing^77,90,91^. In other words, the activity observed in this contrast appears to partially reflect processing other than of age. Moreover, the data we obtained could not be decomposed into activity reflecting age processing and activity reflecting other processing. Thus, we discontinued further analysis of brain activity related to processing of facial age, and focused on facial attractiveness (for other contrasts between different tasks, see Supplementary Fig. S6 online, Supplementary Table S3, S4 online).

## Discussion

### Summary

The mOFC was significantly more active when judging facial attractiveness than when judging facial skin reflection (Fig. 2). This result suggests that the mOFC is involved in processing facial attractiveness that arises from skin reflection. It should be noted that since an identical set of faces were presented in both tasks, difference in activation should not be attributed to differences in the stimulus between the tasks, but to differences in the features that the subjects paid attention to during the tasks (facial attractiveness). In addition, the reason we claim that facial attractiveness processed in the mOFC in our study was based on skin reflection rather than from other cues is that (1) the subjects were asked to judge facial attractiveness solely based on skin impression and (2) that the only differences between the skins in the stimuli were the types of skin reflection (i.e., matte, oily-shiny, or radiant).

In the psychological experiment, rated attractiveness was highest with radiant, then oily-shiny, then matte skin (Fig. 3). Comparison of the results of the fMRI and psychological experiments showed that the level of activation in the mOFC significantly increased with the rated attractiveness (Fig. 4). These results suggest that the activation level in the mOFC reflects facial attractiveness based on skin reflection.

### Involvement of mOFC in processing facial attractiveness from skin reflection

The results of the present fMRI experiment suggest that the mOFC is involved in processing facial attractiveness based on skin reflection. This claim is in line with previous studies reporting that the mOFC is involved in processing facial attractiveness of smiling^54^, cosmetics use^61^, or various other factors that create differences in facial attractiveness between different facial models^53,55,58–60^. Moreover, in the present study, the mOFC activation significantly increased with the rated attractiveness (Fig. 4). Activity in the mOFC has previously been reported to represent or monitor reward values^54,92–99^. Thus, the results of the present study, together with previous reports, could suggest that facial attractiveness from skin reflection is a reward to the beholder.

One could doubt that the mOFC activation observed in this study reflects reward values because the mOFC activation was lower during the tasks than during the rest (the baseline in Fig. 4) in many stimulus conditions. Although we cannot completely dispel this doubt, the reward-value view seems plausible in light of previous studies as described in the prior paragraph. The lower activation in the mOFC during the tasks may be explained by the possible stress; since all the tasks were to be performed very quickly and they also required memorizing and remembering attractiveness, age, or skin reflection of all presented facial images except the last one, the subjects might have been more stressed during the tasks than during the rest.

Contrarily, a few previous studies did not find OFC activation associated with facial attractiveness^63,70,71^. This could at least partially be due to losses of the fMRI signal in the OFC caused by susceptibility gradients near air/tissue interfaces^100–102^. In the present fMRI experiment, to minimize such susceptibility artefacts in the OFC, we optimized MRI-data acquisition parameters, including slice orientation and phase encoding direction^100,101^ (for detail, see “MRI data acquisition” section), as did some of the studies that found activation in the OFC from facial attractiveness^57,58,62^.

Meanwhile, as described in the introductory section, some previous studies also found activations within other human brain regions associated with facial attractiveness, including brain regions in the reward systems as well as regions in the “face network” ^64–68^. Although our fMRI results do not exclude the possibility of the involvement of other such brain regions in processing facial attractiveness from skin reflection, one possible reason why we did not identify activity in these regions in our experiment is that at least some of these regions may not represent facial attractiveness but be involved in other processes. For instance, the ventral striatum has been reported to encode prediction errors of reward values rather than reward values per se^58,103^, and the thalamus has been reported to be involved in the executive control of attention^90,91,104,105^.

It also should be noted that the present study determined the involvement of the mOFC in processing facial attractiveness based on skin reflection by contrasting the activation for judging attractiveness with the activation for judging skin reflection. Hence, activity in brain regions activated in both task periods should have been cancelled out. These regions are likely to be upstream to the mOFC, and may include early visual areas which process low-level visual features, mid-level visual areas involved in processing surface reflection^82,106,107^, and the FFA, which is involved in face-related processing^108,109^.

### Effects of sex, culture, and era

It has been found that the combination of the facial model and the observer's sexes^58^, or the observer's sex preference^110^, affects attractiveness ratings. In the present study, both the models and the observers were females. This begs the question as to whether the mOFC is involved in processing facial attractiveness based on skin reflection with other combinations of sexes. The involvement of the mOFC is likely to hold true in the light of a series of previous studies which reported that the mOFC was activated by facial attraction irrespective of the combination of sexes between the stimulus face and the observer^53,54,57,58,61^. Although mOFC activation magnitudes have been reported to be higher when the model's sex was different to the observer's^59^, or was the observer's preferred sex^55,56^, such sex effects reflect the attractiveness ratings^59^ (but see also others^55,56^, which reported higher mOFC responses to preferred sex even when the attractiveness ratings did not reflect sex preference). Since in the present study, both the models and the observers were of the same sex (female), the mOFC is likely to be involved in processing facial attractiveness based on skin reflection even for other sex combinations, although the attractiveness itself from skin reflection could be somewhat limited if both the face and the observer are male. Effects of sex on facial attractiveness itself will be discussed later.

Another interesting question relates to the attraction of faces which are of the same sex as the observer. According to Franklin & Adams' theory^111^, facial attractiveness incorporates both sexual and aesthetic values. Hence, if the observer is heterosexual, the facial attractiveness of the same sex as the observer seems to be aesthetic value or beauty. Interestingly, the mOFC can also be more activated even when viewing paintings that were rated beautiful compared with those rated neutral or ugly even if the paintings do not contain a face^112^. Therefore, our finding that the mOFC is the neural component underlying judgement of facial attractiveness is quite reasonable even if the facial models and the observers were of the same sex.

Despite the importance of the mOFC in judging facial attractiveness from skin reflection, it should be noted that facial attractiveness itself has been reported to depend not only on health but also on other factors including culture^2^, era^113^, and the sexes of the face^20^ and the observer^27,114,115^. Hence it remains to be seen whether the effects of skin reflection on facial attractiveness reported in the present study are consistent when applied to any face and any observer throughout the world. Such a question may also lead to revealing innate and learned components in the neural mechanism for judging facial attractiveness.

### Why does skin reflection affect facial attractiveness?

In the psychological experiment, rated attractiveness increased from faces with matte, to oily-shiny, to radiant skin. This result is consistent with the findings of our recent study^43^, where we used a pairwise comparison method. Another very recent study has also reported that increases in specular reflection enhance facial attractiveness^116^. The only exception is Arce-Lopera and co-workers^86^, who reported that square patches of cheek image appeared younger when the specular reflection component was removed. Although people say that radiant skin is more desirable than oily-shiny skin or matte skin, to our knowledge, before our recent study mentioned above^43^, there was little scientific research comparing the attractiveness of faces with matte, oily-shiny, and radiant skins.

Why does facial attractiveness increase in this order? Here, we propose three possible explanations. The first explanation is that, as explained in the introduction section, the health condition, including the soundness of the immune system of the skin, seems to be enhanced in this order due to both sebum on the skin (for radiant and oily-shiny skin) and moisture in the skin (for the radiant skin). Hence, the facial attractiveness based on skin reflection is likely to indicate the degree of health, in line with facial attractiveness from other factors^1,2^. This first explanation also includes possible relationships between the healthiness and (1) luminance uniformity over the skin and (2) fine textures. Radiant skin generally has more luminance uniformity^31,49,117^ and finer texture^29–31^ than those of oily-shiny skin. Luminance uniformity over the skin^17,22^ and texture fineness^118^ have been reported to enhance facial attractiveness. The texture fineness also has been reported to enhance perceived health^18^. Thus, luminance uniformity and fine textures could also reflect good health condition, thereby enhancing facial attractiveness.

The second explanation, which is compatible with the first one, is that such skin reflection types indicate skin age. That is, facial attractiveness from the skin reflection may imply the skin age. This explanation is supported by and/or consistent with several lines of evidence. First, in the psychological experiment on age in the present study, the rated age decreased from matte to oily-shiny to radiant skin (Supplementary Fig. S3 online), just as the attractiveness increased in this order (Fig. 3). Moreover, the rated attractiveness was negatively correlated with the rated age (Supplementary Fig. S4 online), just as the female facial attractiveness based on any available cue decreases with the real age of the face^87,115,119–121^. Second, again, the luminance uniformity^122,123^ and texture fineness, which generally exist on radiant skin, decrease with age^124^. Third, diffuse reflection, which generally exists on the radiant skin more than on the oily-shiny skin^30^, decreases with age^30^.

The third explanation involves visual illusory effects in which the skin reflection enhances the impression of smiling. Smiling has been reported to enhance facial attractiveness^15^. When smiling, the cheeks generally become more convex. Meanwhile, the specular highlights on the cheeks cause an illusion of the cheeks being more convex (Fig. 1), as has been reported on the surfaces of non-skin objects^125^. Hence, such an illusion may appear as more of a smile, as can be seen in Fig. 1.

Conversely, the effect of smiling on facial attractiveness, which has been reported previously^15^, could (at least partially) be because of the shine on the cheeks which is enhanced by real convexity due to a real smile (but see also Kampe et al., 2001^16^, who pointed out the effects of gaze direction). That is, smiling causes the cheeks to be more convex, thereby illusorily enhancing impression of shine on the cheeks (as has been reported to be the case with non-skin surfaces^126^), which may be a sign of good health as described above as the first explanation.

Although these three explanations (as well as the one mentioned in the previous paragraph) are possible, further study is required to validate them.

It should be noted that the explanations based on health and age mentioned above assume that the skin reflection is naturally yielded by the skin itself. On the other hand, we used cosmetic materials to control the conditions of skin reflection for the stimulus images (for details, see "Stimuli" subsection). Hence, the reflection was not wholly yielded by the skin itself, thereby limiting the validity of the claim that findings were based on health and age. However, we believe that these explanations would still be possible due to the following two reasons. First, the naturalness of appearance of the skin reflection in the images was confirmed by many people, including those who evaluated facial photos as skilled professionals (for details, see Supplementary Methods online). Second, as mentioned above, the explanation based on health is consistent with numerous previous studies on facial attractiveness. In a similar vein, the explanation based on age is consistent with several lines of evidence mentioned above.

### Research on surface reflection properties

It is noteworthy that recent developments in the field of computer graphics technologies have enabled researchers to explore the mechanisms of perception of object material properties, especially surface reflection properties (i.e., glossiness) ^82,106,107,126–134^. Despite an extensive body of literature on the perception of surface reflection, little is known about its effects on higher cognition. In this context, the present study provides not only psychological but also neuroscientific evidence for the impact of surface reflection properties on higher cognition, especially, on facial attractiveness.

### Limitations and future directions

Here we summarize the present study's limitations and the future directions.

It remains to be seen whether the observer's sex and sexual orientation as well as the facial model's sex affect the effects of skin reflection on the facial attractiveness and the brain activities. This is because in the present study, (1) all the subjects and the models were females and 2) the sexual orientations of two of the sixteen subjects are unknown, whereas the other subjects reported that they were heterosexual. In addition, while in our previous^43^ and the present studies, both the subjects and the facial models were in their 30s to 40s, their ages could also affect the results.

Moreover, further study is required to determine (1) why the skin reflection enhances facial attractiveness, (2) whether the cortical activation elicited by skin reflection reflects a reward to the observer, and (3) the entire neural mechanism by which facial skin reflection enhances facial attractiveness.

## Conclusion

The present study demonstrated that skin reflection enhances facial attractiveness and that such attractiveness is reflected by the activation level of the human frontal cortex, especially the medial part of the orbitofrontal cortex (mOFC). Since facial attractiveness based on skin reflection might reflect health^1,2^, measurement of the level of activation of the mOFC may be of help for the estimation of not only facial attractiveness but also the health of an individual. Finally, the present fMRI and psychological results, together with previous reports, suggest that conditioning the facial skin by using cosmetics may aid women to become more attractive^75,113,114^ and more rewarding to the beholders^61^ not only by increasing luminance contrast between the eyes, lips, and skin^20,21,26^_,_ but also by naturally enhancing skin reflection which gives the implication of a good condition of health.

## Methods

### Subjects

Eighteen females (32–49 years of age, mean 39.9) naive to the purpose of the study took part in both the fMRI and the psychological experiments. To avoid bias in favor of skin reflection, the subjects were recruited via a temporary employment agency, who did not know the purpose of the present study. In addition, the subjects and their families did not work for companies related to cosmetics, toiletries, or mass media including cosmetic magazines. All subjects lived in Japan and their native language was Japanese. They were healthy with no history of neurological disorders, had normal or corrected-to-normal vision, and gave written informed consent before each experiment. The experimental procedures were approved by the ethics, the personal data, and the safety committees of the National Institute of Information and Communication Technology and by the Ethical Committee of the Shiseido Global Innovation Center. All methods were performed in accordance with the relevant guidelines and regulations. Data from two subjects were excluded from the analysis because they did not follow the instructions when conducting the tasks. In a post hoc questionnaire, fourteen out of all the sixteen subjects whose data were included in the analysis reported that they were heterosexual while the other two refrained from reporting their sexual orientations.

### Apparatus

We used a Siemens 3-T MAGNETOM Prisma scanner to conduct the fMRI experiment. The subjects lay in the scanner and were exposed to the stimulus images presented on a 24-inch MRI-compatible liquid crystal display (1920 × 1200, 518.4 mm × 324.0 mm, 60 Hz, BOLDscreen 24 LCD for fMRI, Cambridge Research Systems, UK) located adjacent to the scanner. The viewing distance was 125 cm via a mirror. Using a photometer (i1 Publish Pro2, X-Rite, USA), we adjusted the maximum luminance and the gamma value to 68 cd/m^2^ and 2.2, respectively. To control the stimulus presentation, we used an original C program with OpenGL running on a personal computer (HP Z840 Workstation). To perform the tasks, the subjects pressed buttons on an MRI-compatible button box (HHSC-2X2, Current Designs, USA).

In the psychological experiment, the subjects were seated on a chair in a dark room where they were exposed to the stimulus images presented on an identical display to the one used in the fMRI experiment. The viewing distance and the methods to control the stimulus presentation were the same as those in the fMRI experiment.

### Stimuli

In both the fMRI and the psychological experiments, we used female facial images with matte (Fig. 1a), oily-shiny (Fig. 1b), and radiant (Fig. 1c) skin. The faces were of nine Japanese female models (30–44 years of age) unfamiliar to the subjects. The whole process of preparing the images involved two stages. During the photo-taking, photos of the models' faces with several different cosmetic materials were taken under different lighting conditions. All makeup except for skincare was manipulated by a professional makeup artist. Photos were then retouched using computer graphics techniques to generate facial images with matte, oily-shiny, or radiant skins. We took previous studies^30,31,49^ into consideration to prepare the stimulus images (for more detail of the whole process of image preparation, see Supplementary Methods online). The size of the final images used in the experiments was 719 (W) × 1078 (H) pixels (8.9 deg × 13.3 deg in visual angle). In both the fMRI experiment and the two psychological experiments (one centred around attractiveness and one around age), the images of eight models were used, each with all three skin reflection types, resulting in a total of 24 images. Dummy facial images for the fMRI experiment described in the next section were also chosen randomly from within them. In the other psychological experiment into reflection, the same 24 images, as well as an image of the ninth model, who had only oily-shiny skin, were used as test stimuli and a standard stimulus, respectively. All models pictured in this manuscript and the  supplementary information have given informed consent to publish their facial images in an online open-access publication.

### Procedure and tasks

#### fMRI experiment

We presented stimuli in an event-related mini-block design (adapted from Wurm et al., 2016^135^; Supplementary Fig. S1 online). As described later in the section about fMRI Data Analysis, we used a block design to examine the effects of task induced attention while an event-related design was used to examine the effects of stimuli.

Before each task block, a task attribute (“attractiveness”, “age”, or “reflection”) was presented for 2 s, followed by a 1-s fixation period (Supplementary Fig. S1 online). In each block, 13 randomly chosen face images, including the first dummy image, were each presented successively for 2 s with a 1 s inter-stimulus interval (ISI). The subjects were asked to judge whether each face rated higher or lower than the immediately preceding face in terms of the task attribute presented before the block, based solely on skin quality rather than other factors such as facial shape (i.e., 1-back task). They were asked to press one of the two buttons that corresponded to their judgement using their right thumb as soon as each facial image disappeared and a white circle appeared. Specifically, the left and the right button corresponded to a judgement of “lower” and “higher”, respectively.

In each scanning run, two blocks were performed for each task in a randomized order, resulting in a total of six blocks. All 24 stimuli were presented in the two blocks of the same task in each run, apart from the first dummy image of each block. Importantly, the identical set of 24 stimulus images was presented for all three different tasks. This was so that the differences in the brain activation between the different tasks could be attributed to differences in the performed task rather than differences in stimulus. Such orthogonal design was also crucial to avoid “double dipping”, an invalid statistical inference that can arise when the same data is used for both identification of the region of interest (ROI) by the inter-task comparison and ROI-wise analyses by the inter-stimulus comparison^136^. How this issue was avoided will be explained later in the fMRI Data Analysis section. The dummy faces were 16 images that were randomly chosen for each subject from within all the 24 facial images so that different dummy faces were applied randomly to all the 16 blocks of each task. Importantly, identical 16 dummy faces were presented in all different tasks for each subject (Wurm et al., 2016). Each dummy face was different from the subsequent face for the first trial of each block (Supplementary Fig. S1). Successive blocks were interleaved with a 12 s fixation period, as well as the 2 s task attribute and the 1 s fixation period described above. Each run started with a 10 s fixation period and ended with a 16 s fixation period. During each run, the subjects were asked to fixate the centre of the display. Eight runs were carried out for each subject.

Before the fMRI experiment, the subjects were given instructions and practiced performing 36 trials of the tasks in a waiting room. They then practiced one whole run of 72 trials in the scanner during the anatomical scan. In the instructions, the experimenter explicitly explained that the definition of “reflection” was “the magnitude of reflection of light in one direction from the surface” and showed the illustration of surface reflection, which corresponds to surface glossiness (Supplementary Fig. S2 online). In the debriefing after the fMRI experiment, the experimenter confirmed that all subjects (except the two mentioned in the Subjects section) performed the tasks according to the instructions.

#### Psychological experiments

At a later date, the psychological experiments were conducted to examine the subjective absolute (rather than relative) magnitudes of attractiveness, age, and reflection of each facial image used in the fMRI experiment. These subjective magnitudes were measured so that they could be compared with fMRI signal intensity (i.e. % change in BOLD signal) of the brain regions involved in processing those attributes.

In sessions for the attractiveness evaluation experiment, all 24 facial images used in the fMRI experiment were successively presented for 2 s each in a randomized order. The subjects were asked to rate each face's attractiveness on a scale of 1 (“very unattractive”), 2 (“unattractive”), 3 (“neutral”), 4 (“attractive”), and 5 (“very attractive”)^53,55,111^ and made an oral report while a subsequent white circle was presented for 4 s. Similarly, in an age estimation experiment, the same 24 images were successively presented for 2 s each in a randomized order. The subjects were asked to report the estimated age during a 5 s period whilst the white circle was displayed. In a reflection rating experiment, the standard and the test stimulus were presented successively for 2 s each with an ISI of 1 s. Each test stimulus was one of the 24 images used in the fMRI experiment. In a subsequent 4 s white circle period, the subjects were asked to report the magnitude of the specular reflection from the whole facial skin, giving a number based on the understanding that the specular reflection of the standard stimulus was ten (magnitude estimation method). Zero meant no specular reflection (completely matte). The subjects were explicitly allowed to report any number that was equal to or higher than zero, including ten and higher numbers. This reflection task corresponds to reporting perceived surface glossiness^128,129^. In all these psychological experiments, the subjects were asked to report solely based on skin quality as in the fMRI experiment. Three sessions (i.e. repetitions) were carried out for each experiment and the three experiments of different tasks were conducted in a randomized order for each subject.

As in the fMRI experiment, before each psychological experiment, the subjects were given instructions and observed all the stimuli for a whole run of 24 trials, and practiced reporting in the first 10 trials. Then they practiced performing tasks of a whole run. In the instructions, the experimenter again explicitly explained the definition of “reflection” in exactly the same way as during the fMRI experiment. In the debriefing after each psychological experiment, the experimenter confirmed that all of the subjects (except the two mentioned in the Subjects section) performed the tasks according to the instructions.

### MRI data acquisition

Structural and functional MRI data were collected using a 64-channel head coil. We acquired fMRI data of the whole brain using a multiband gradient echo-planar imaging sequence^137^ with the following imaging parameters: repetition time (TR) = 2,000 ms, echo time (TE) = 28 ms, flip angle (FA) = 75 deg, voxel size = 2 mm × 2 mm × 2 mm, matrix size = 96 × 96, 75 slices, no gaps between slices, slice tilt = 20 deg (anterior upwards) from the anterior–posterior commissure (AC–PC) line, phase-encoding direction from anterior to posterior, slice order of interleaved increasing, multi-band factor = 3, iPAT factor = 2. These parameters, specifically, the slice thinness, slice orientation, and phase-encoding direction were chosen based on pilot scan data as well as previous reports^100–102^ in order to minimize susceptibility artefacts in the OFC. We also acquired T1-weighted anatomical images of the whole brain with the following imaging parameters: TR = 1,900 ms, TE = 3.37 ms, FA = 9 deg, voxel size = 1 mm × 1 mm × 1 mm, matrix size = 256 × 256, 208 slices.

### fMRI data analysis

We used SPM12 software (http://www.fil.ion.ucl.ac.uk/spm) to carry out statistical parametric mapping (SPM) analysis of the whole-brain fMRI data from all 16 of the subjects that followed the instructions during the experiments. We applied the following pre-processing steps to the functional EPI images: slice-timing correction, motion correction, registration with anatomical images, normalization to the Montreal Neurological Institute (MNI) stereotaxic space, and spatial smoothing using a 3D Gaussian kernel with a 4-mm full width at half maximum. We conducted SPM analysis based on the general linear model (GLM).

Our fMRI experiment simultaneously employed both a block design and an event-related design to examine in one experiment the effects of tasks and those of stimuli, respectively. As described above, we used the same set of stimuli in the different task conditions as per a previous study^135^. This design was essential to avoid double dipping, a statistically invalid inference that can be caused by using the same data both for localizing an ROI and for extracting responses within the obtained ROI^136^. We orthogonalized the effects of tasks and those of stimuli by using the following analyses, which enabled us to independently examine each of these effects.

To examine the effects of tasks, each of the three task conditions (i.e., attractiveness, age, and skin reflection) was modelled as an independent GLM regressor in each run. The six-dimensional head-motion correction parameters were also incorporated as regressors in the model to isolate effects of subjects' head movements. By conducting the individual-level GLM analysis, a set of beta-coefficient images corresponding to the regressors were generated. These images were used for the subsequent group-level analysis that examined all the six contrasts between every two of the three tasks (e.g. attractiveness > skin reflection). To the resulting activation maps, we then applied both an uncorrected peak-level threshold of *p* < 0.001, and a multiple-comparison correction with a cluster-level false discovery rate (FDR) set at *p* < 0.05. The regions of the peak voxels of the resulting clusters were identified by using the automated anatomical labeling (AAL) atlas^88^. The Brodmann areas of the peak voxels were determined by using Yale BioImage Suite Package^138^ (http://sprout022.sprout.yale.edu/mni2tal/mni2tal.html).

To examine the effects of the stimuli on the activity in the regions involved in processing attractiveness based on skin reflection, we used the MarsBaR toolbox for SPM (http://marsbar.sourceforge.net/). We first identified an ROI (consequently, the mOFC) from the contrast of “attractiveness > skin reflection” in the between-task analysis described above. Using the same toolbox, we then calculated the beta-coefficient averaged across all voxels within the ROI for each of all the 24 stimulus conditions, and finally, from this beta-coefficient, we calculated the BOLD percent signal change (i.e., the level of activation in the mOFC).

## Supplementary Information

Supplementary Information.

## References

[1] Thornhill R, Gangestad SW (1999). Facial attractiveness. Trends Cogn. Sci..

[2] Little, A. C., Jones, B. C. & DeBruine, L. M. Facial attractiveness: evolutionary based research. *Philos. Trans. R. Soc. Lond. B Biol. Sci.* **366**, 1638–1659, 10.1098/rstb.2010.0404 (2011). 10.1098/rstb.2010.0404 PMC3130383 21536551

[3] Buss DM, Barnes M (1986). Preferences in human mate selection. J. Pers. Soc. Psychol..

[4] Cash TF, Kilcullen RN (1985). The aye of the beholder: Susceptibility to sexism and beautyism in the evaluation of managerial applicants. J. Appl. Soc. Psychol..

[5] Chiu RK, Babcock RD (2002). The relative importance of facial attractiveness and gender in Hong Kong selection decisions. Int. J. Hum. Resour. Manag..

[6] Langlois JH, Roggman LA (1990). Attractive faces are only average. Psychol. Sci..

[7] Thornhill R, Gangestad SW (1993). Human facial beauty: Averageness, symmetry, and parasite resistance. Hum. Nat..

[8] Perrett DI, May KA, Yoshikawa S (1994). Facial shape and judgements of female attractiveness. Nature.

[9] Baudouin JY, Tiberghien G (2004). Symmetry, averageness, and feature size in the facial attractiveness of women. Acta Psychol. (Amst.).

[10] Valentine T, Darling S, Donnelly M (2004). Why are average faces attractive? The effect of view and averageness on the attractiveness of female faces. Psychon. Bull. Rev..

[11] DeBruine LM, Jones BC, Unger L, Little AC, Feinberg DR (2007). Dissociating averageness and attractiveness: Attractive faces are not always average. J. Exp. Psychol. Hum. Percept. Perform..

[12] Damon F (2017). Preference for facial averageness: Evidence for a common mechanism in human and macaque infants. Sci. Rep..

[13] Tomeo OB, Ungerleider LG, Liu N (2017). Preference for averageness in faces does not generalize to non-human primates. Front. Behav. Neurosci..

[14] Perrett DI (1998). Effects of sexual dimorphism on facial attractiveness. Nature.

[15] Otta, E., Folladore Abrosio, F. & Hoshino, R. L. Reading a smiling face: Messages conveyed by various forms of smiling. *Percept. Mot. Skills* **82**, 1111–1121, 10.2466/pms.1996.82.3c.1111 (1996). 10.2466/pms.1996.82.3c.1111 8823879

[16] Kampe KK, Frith CD, Dolan RJ, Frith U (2001). Reward value of attractiveness and gaze. Nature.

[17] Fink B, Grammer K, Thornhill R (2001). Human (Homo sapiens) facial attractiveness in relation to skin texture and color. J. Comp. Psychol..

[18] Fink B, Matts PJ (2008). The effects of skin colour distribution and topography cues on the perception of female facial age and health. J. Eur. Acad. Dermatol. Venereol..

[19] Fink B (2018). Hair color and skin color together influence perceptions of age, health and attractiveness in lightly pigmented young women. Int. J. Cosmet. Sci..

[20] Russell R (2003). Sex, beauty, and the relative luminance of facial features. Perception.

[21] Russell R (2009). A sex difference in facial contrast and its exaggeration by cosmetics. Perception.

[22] Matts PJ, Fink B, Grammer K, Burquest M (2007). Color homogeneity and visual perception of age, health, and attractiveness of female facial skin. J. Am. Acad. Dermatol..

[23] Stephen, I. D., Law Smith, M. J., Stirrat, M. R. & Perrett, D. I. Facial skin coloration affects perceived health of human faces. *Int. J. Primatol.* **30**, 845–857, 10.1007/s10764-009-9380-z (2009). 10.1007/s10764-009-9380-z PMC2780675 19946602

[24] Stephen ID, Coetzee V, Law Smith M, Perrett DI (2009). Skin blood perfusion and oxygenation colour affect perceived human health. PLoS ONE.

[25] Stephen ID, McKeegan AM (2010). Lip colour affects perceived sex typicality and attractiveness of human faces. Perception.

[26] Etcoff NL, Stock S, Haley LE, Vickery SA, House DM (2011). Cosmetics as a feature of the extended human phenotype: Modulation of the perception of biologically important facial signals. PLoS ONE.

[27] Tani Y (2018). The concept of the attractiveness of the skin. Cosmetology.

[28] Doi, H., Tsumura, N. & Shinohara, K. Neural basis of attractiveness perception from facial skin color. *J. Fac. Stud.* **17**(1), 30–30 (2017) **(abstract in Japanese)**.

[29] Masuda, Y., Kunizawa, N. & Takahashi, M. Methodology for evaluation of skin transparency and the efficacy of an essence that can improve skin transparency. *J. Soc. Cosmet. Chem. Jpn.* **39**, 201–208, 10.5107/sccj.39.3_201 (2005) **(in Japanese)**.

[30] Masuda, Y., Yagi, E., Oguri, M. & Kuwahara, T. Development of a quantitative method for evaluation of skin radiance and its relationship with skin surface topography. *J. Soc. Cosmet. Chem. Jpn.* **51**, 211–218, 10.5107/sccj.51.211 (2017) **(in Japanese)**.

[31] Fujii, M., Misaki, Y. & Sasaki, I. Application of image processing technique for facial gloss evaluation. *J. Soc. Cosmet. Chem. Jpn.* **43**, 72–78, 10.5107/sccj.43.72 (2009) **(in Japanese)**.

[32] Rawlings AV, Harding CR (2004). Moisturization and skin barrier function. Dermatol. Ther..

[33] Boer M, Duchnik E, Maleszka R, Marchlewicz M (2016). Structural and biophysical characteristics of human skin in maintaining proper epidermal barrier function. Postepy Dermatol. Alergol..

[34] Purnamawati S, Indrastuti N, Danarti R, Saefudin T (2017). The role of moisturizers in addressing various kinds of dermatitis: A review. Clin. Med. Res..

[35] Elias PM (2007). The skin barrier as an innate immune element. Semin. Immunopathol..

[36] Zlotogorski A (1987). Distribution of skin surface pH on the forehead and cheek of adults. Arch. Dermatol. Res..

[37] Rippke F, Schreiner V, Schwanitz HJ (2002). The acidic milieu of the horny layer: new findings on the physiology and pathophysiology of skin pH. Am. J. Clin. Dermatol..

[38] Schmid-Wendtner MH, Korting HC (2006). The pH of the skin surface and its impact on the barrier function. Skin Pharmacol. Physiol..

[39] Schmid-Wendtner, M. & Korting, H. C. *pH and Skin Care*. 43–45 (ABW Wissenschaftsverlag, 2007).

[40] Drake DR, Brogden KA, Dawson DV, Wertz PW (2008). Antimicrobial lipids at the skin surface. J. Lipid Res..

[41] Ali SM, Yosipovitch G (2013). Skin pH: From basic science to basic skin care. Acta Derm. Venereol..

[42] Porter AM (2001). Why do we have apocrine and sebaceous glands?. J. R. Soc. Med..

[43] Ikeda *et al.* Facial radiance influences facial attractiveness and affective impressions of faces. *Int. J. Cosmet. Sci.*10.1111/ics.12673 (2020). 10.1111/ics.12673 PMC8246902 33217010

[44] Musnier, C., Piquemal, P., Beau, P. & Pittet, J. C. Visual evaluation in vivo of 'complexion radiance' using the C.L.B.T. sensory methodology. *Skin Res. Technol.* **10**, 50–56 (2004). 10.1111/j.1600-0846.2004.00053.x 14731249

[45] Baret M (2006). Characterization and quantification of the skin radiance through new digital image analysis. Skin Res. Technol..

[46] Petitjean A (2007). Skin radiance: How to quantify? Validation of an optical method. Skin Res. Technol..

[47] Matsubara A (2012). Differences in the surface and subsurface reflection characteristics of facial skin by age group. Skin Res. Technol..

[48] Matsubara A, Liang Z, Sato Y, Uchikawa K (2012). Analysis of human perception of facial skin radiance by means of image histogram parameters of surface and subsurface reflections from the skin. Skin Res. Technol..

[49] Ohtsuki R, Hikima R, Sakamaki T, Tominaga S (2013). Evaluation method of oily-shine using facial image with cosmetics foundation. J. Color Sci. Assoc. Jpn..

[50] Mizukoshi K, Akamatsu H (2013). The investigation of the skin characteristics of males focusing on gender differences, skin perception, and skin care habits. Skin Res. Technol..

[51] Igarashi T, Nishino K, Nayar SK (2007). The appearance of human skin: A survey. Found. Trends Comput. Graph. Vis..

[52] Nakamura K (1998). Neuroanatomical correlates of the assessment of facial attractiveness. NeuroReport.

[53] Aharon I (2001). Beautiful faces have variable reward value: fMRI and behavioral evidence. Neuron.

[54] O'Doherty J (2003). Beauty in a smile: The role of medial orbitofrontal cortex in facial attractiveness. Neuropsychologia.

[55] Kranz F, Ishai A (2006). Face perception is modulated by sexual preference. Curr. Biol..

[56] Ishai A (2007). Sex, beauty and the orbitofrontal cortex. Int. J. Psychophysiol..

[57] Winston JS, O'Doherty J, Kilner JM, Perrett DI, Dolan RJ (2007). Brain systems for assessing facial attractiveness. Neuropsychologia.

[58] Bray S, O'Doherty J (2007). Neural coding of reward-prediction error signals during classical conditioning with attractive faces. J. Neurophysiol..

[59] Cloutier J, Heatherton TF, Whalen PJ, Kelley WM (2008). Are attractive people rewarding? Sex differences in the neural substrates of facial attractiveness. J. Cogn. Neurosci..

[60] Tsukiura T, Cabeza R (2011). Remembering beauty: Roles of orbitofrontal and hippocampal regions in successful memory encoding of attractive faces. Neuroimage.

[61] Ueno A (2014). Neural activity associated with enhanced facial attractiveness by cosmetics use. Neurosci. Lett..

[62] Shen H (2016). Brain responses to facial attractiveness induced by facial proportions: Evidence from an fMRI study. Sci. Rep..

[63] Chatterjee A, Thomas A, Smith SE, Aguirre GK (2009). The neural response to facial attractiveness. Neuropsychology.

[64] Ishai A (2008). Let's face it: It's a cortical network. Neuroimage.

[65] Wiggett AJ, Downing PE (2008). The face network: Overextended? (Comment on: "Let's face it: It's a cortical network" by Alumit Ishai). Neuroimage.

[66] Rossion B (2008). Constraining the cortical face network by neuroimaging studies of acquired prosopagnosia. Neuroimage.

[67] Tsao DY, Moeller S, Freiwald WA (2008). Comparing face patch systems in macaques and humans. Proc. Natl. Acad. Sci. U. S. A..

[68] Barraclough, N. E. & Perrett, D. I. From single cells to social perception. *Philos. Trans. R. Soc. Lond. B Biol. Sci.* **366**, 1739–1752, 10.1098/rstb.2010.0352 (2011). 10.1098/rstb.2010.0352 PMC3130376 21536557

[69] Kim H, Adolphs R, O'Doherty JP, Shimojo S (2007). Temporal isolation of neural processes underlying face preference decisions. Proc. Natl. Acad. Sci. U. S. A..

[70] Bzdok D (2012). The modular neuroarchitecture of social judgments on faces. Cereb. Cortex.

[71] Kedia G, Mussweiler T, Mullins P, Linden DE (2014). The neural correlates of beauty comparison. Soc. Cogn. Affect. Neurosci..

[72] Halit H, de Haan M, Johnson MH (2000). Modulation of event-related potentials by prototypical and atypical faces. NeuroReport.

[73] Trujillo LT, Jankowitsch JM, Langlois JH (2014). Beauty is in the ease of the beholding: A neurophysiological test of the averageness theory of facial attractiveness. Cogn. Affect. Behav. Neurosci..

[74] Hahn AC (2016). Early and late event-related potentials are modulated by infant and adult faces of high and low attractiveness. Soc. Neurosci..

[75] Tagai K, Shimakura H, Isobe H, Nittono H (2017). The light-makeup advantage in facial processing: Evidence from event-related potentials. PLoS ONE.

[76] Corbetta M, Miezin FM, Dobmeyer S, Shulman GL, Petersen SE (1990). Attentional modulation of neural processing of shape, color, and velocity in humans. Science.

[77] Liu T, Slotnick SD, Serences JT, Yantis S (2003). Cortical mechanisms of feature-based attentional control. Cereb. Cortex.

[78] Murray SO, Wojciulik E (2004). Attention increases neural selectivity in the human lateral occipital complex. Nat. Neurosci..

[79] Peuskens H (2004). Attention to 3-D shape, 3-D motion, and texture in 3-D structure from motion displays. J. Cogn. Neurosci..

[80] Cant JS, Goodale MA (2007). Attention to form or surface properties modulates different regions of human occipitotemporal cortex. Cereb. Cortex.

[81] Cant JS, Goodale MA (2011). Scratching beneath the surface: New insights into the functional properties of the lateral occipital area and parahippocampal place area. J. Neurosci..

[82] Wada A, Sakano Y, Ando H (2014). Human cortical areas involved in perception of surface glossiness. Neuroimage.

[83] Kite ME, Stockdale GD, Whitley BE, Johnson BT (2005). Attitudes toward younger and older adults: an updated meta-analytic review. J. Soc. Issues.

[84] Rhodes MG (2009). Age estimation of faces: A review. Appl. Cogn. Psychol..

[85] Eiamkanchanalai, S., Assarut, N. & Surasiengsunk, S. Attitude toward the elderly and social interaction: Approach toward an intergenerational society. *Kasetsart J. Soc. Sci.*10.1016/j.kjss.2017.12.010 (2018) **(in press)**.

[86] Arce-Lopera C, Igarashi T, Nakao K, Okajima K (2012). Effects of diffuse and specular reflections on the perceived age of facial skin. Opt. Rev..

[87] Nagasaki, F. & Murakami, M. Influential factors of age perception from the facial image of women in their 30s and 40s. *J. Soc. Cosmet. Chem. Jpn.* **50**, 17–24, 10.5107/sccj.50.17 (2016) **(in Japanese)**.

[88] Tzourio-Mazoyer N (2002). Automated anatomical labeling of activations in SPM using a macroscopic anatomical parcellation of the MNI MRI single-subject brain. Neuroimage.

[89] Homola GA, Jbabdi S, Beckmann CF, Bartsch AJ (2012). A brain network processing the age of faces. PLoS ONE.

[90] Yantis S, Serences JT (2003). Cortical mechanisms of space-based and object-based attentional control. Curr. Opin. Neurobiol..

[91] Petersen SE, Posner MI (2012). The attention system of the human brain: 20 years after. Annu. Rev. Neurosci..

[92] O'Doherty J, Kringelbach ML, Rolls ET, Hornak J, Andrews C (2001). Abstract reward and punishment representations in the human orbitofrontal cortex. Nat. Neurosci..

[93] O'Doherty JP (2004). Reward representations and reward-related learning in the human brain: Insights from neuroimaging. Curr. Opin. Neurobiol..

[94] Kringelbach ML (2005). The human orbitofrontal cortex: Linking reward to hedonic experience. Nat. Rev. Neurosci..

[95] Tsukiura T, Cabeza R (2008). Orbitofrontal and hippocampal contributions to memory for face-name associations: The rewarding power of a smile. Neuropsychologia.

[96] Lebreton M, Jorge S, Michel V, Thirion B, Pessiglione M (2009). An automatic valuation system in the human brain: Evidence from functional neuroimaging. Neuron.

[97] Rushworth, M. F., Noonan, M. P., Boorman, E. D., Walton, M. E. & Behrens, T. E. Frontal cortex and reward-guided learning and decision-making. *Neuron* **70**, 1054–1069, 10.1016/j.neuron.2011.05.014 (2011) 10.1016/j.neuron.2011.05.014 21689594

[98] Chikazoe J, Lee DH, Kriegeskorte N, Anderson AK (2014). Population coding of affect across stimuli, modalities and individuals. Nat. Neurosci..

[99] Berridge KC, Kringelbach ML (2015). Pleasure systems in the brain. Neuron.

[100] Deichmann R, Gottfried JA, Hutton C, Turner R (2003). Optimized EPI for fMRI studies of the orbitofrontal cortex. Neuroimage.

[101] Weiskopf, N., Hutton, C., Josephs, O. & Deichmann, R. Optimal EPI parameters for reduction of susceptibility-induced BOLD sensitivity losses: a whole-brain analysis at 3 T and 1.5 T. *Neuroimage* **33**, 493–504, 10.1016/j.neuroimage.2006.07.029 (2006). 10.1016/j.neuroimage.2006.07.029 16959495

[102] Weiskopf N, Hutton C, Josephs O, Turner R, Deichmann R (2007). Optimized EPI for fMRI studies of the orbitofrontal cortex: Compensation of susceptibility-induced gradients in the readout direction. MAGMA.

[103] Hare TA, O'Doherty J, Camerer CF, Schultz W, Rangel A (2008). Dissociating the role of the orbitofrontal cortex and the striatum in the computation of goal values and prediction errors. J. Neurosci..

[104] Dosenbach NU (2007). Distinct brain networks for adaptive and stable task control in humans. Proc. Natl. Acad. Sci. U. S. A..

[105] Fischer J, Whitney D (2012). Attention gates visual coding in the human pulvinar. Nat. Commun..

[106] Sun HC, Ban H, Di Luca M, Welchman AE (2015). fMRI evidence for areas that process surface gloss in the human visual cortex. Vis. Res..

[107] Sun HC (2016). Differential processing of binocular and monocular gloss cues in human visual cortex. J. Neurophysiol..

[108] Kanwisher N, McDermott J, Chun MM (1997). The fusiform face area: A module in human extrastriate cortex specialized for face perception. J. Neurosci..

[109] Grill-Spector K, Knouf N, Kanwisher N (2004). The fusiform face area subserves face perception, not generic within-category identification. Nat. Neurosci..

[110] Hahn AC, Fisher CI, DeBruine LM, Jones BC (2016). Sex-specificity in the reward value of facial attractiveness. Arch. Sex. Behav..

[111] Franklin RG, Adams RB (2009). A dual-process account of female facial attractiveness preferences: Sexual and nonsexual routes. J. Exp. Soc. Psychol..

[112] Kawabata H, Zeki S (2004). Neural correlates of beauty. J. Neurophysiol..

[113] Tagai K, Ohtaka H, Nittono H (2016). Faces with light makeup are better recognized than faces with heavy makeup. Front. Psychol..

[114] Mulhern R, Fieldman G, Hussey T, Leveque JL, Pineau P (2003). Do cosmetics enhance female Caucasian facial attractiveness?. Int. J. Cosmet. Sci..

[115] Maestripieri D, Klimczuk AC, Traficonte DM, Wilson MC (2014). A greater decline in female facial attractiveness during middle age reflects women's loss of reproductive value. Front. Psychol..

[116] Yasumori, H., Saegusa, C., Okiyama, N. & Kurotani, N. Difference in effects of skin gloss on facial impression perception depending on facial features. *J. Color Sci. Assoc. Jpn.* **42**(6), 56–57, 10.15048/jcsaj.42.6__56 (2018) **(in Japanese)**.

[117] Ojima, N., Haneishi, H. & Miyake, Y. The appearance of skin with make-up (II): Analysis on surface topography of skin with make-up. *Bull. Soc. Sci. Photogr. Jpn.* **56**, 264–269, 10.11454/photogrst1964.56.264 (1993) **(in Japanese)**.

[118] Samson N, Fink B, Matts PJ, Dawes NC, Weitz S (2010). Visible changes of female facial skin surface topography in relation to age and attractiveness perception. J. Cosmet. Dermatol..

[119] McLellan B, McKelvie SJ (1993). Effects of age and gender on perceived facial attractiveness. Can. J. Behav. Sci..

[120] Ishi H, Gyoba J, Kamachi M, Mukaida S, Akamatsu S (2004). Analyses of facial attractiveness on feminised and juvenilised faces. Perception.

[121] Foos PW, Clark MC (2011). Adult age and gender differences in perceptions of facial attractiveness: Beauty is in the eye of the older beholder. J. Genet. Psychol..

[122] Porcheron A, Mauger E, Russell R (2013). Aspects of facial contrast decrease with age and are cues for age perception. PLoS ONE.

[123] Arce-Lopera C, Igarashi T, Nakao K, Okajima K (2013). Image statistics on the age perception of human skin. Skin Res. Technol..

[124] Masuda Y, Oguri M, Morinaga T, Hirao T (2014). Three-dimensional morphological characterization of the skin surface micro-topography using a skin replica and changes with age. Skin Res. Technol..

[125] Mooney SW, Anderson BL (2014). Specular image structure modulates the perception of three-dimensional shape. Curr. Biol..

[126] Nishida, S. & Shinya, M. Use of image-based information in judgments of surface-reflectance properties. *J. Opt. Soc. Am. A Opt. Image Sci. Vis.* **15**, 2951–2965 (1998). 10.1364/josaa.15.002951 9857525

[127] Motoyoshi I, Nishida S, Sharan L, Adelson EH (2007). Image statistics and the perception of surface qualities. Nature.

[128] Sakano Y, Ando H (2010). Effects of head motion and stereo viewing on perceived glossiness. J. Vis..

[129] Sakano Y, Ando H (2012). Psychophysical evaluations of a current multi-view 3-D display: Its advantages in glossiness reproduction. J. Soc. Inf. Display.

[130] Maloney LT, Brainard DH (2010). Color and material perception: Achievements and challenges. J. Vis..

[131] Doerschner K (2011). Visual motion and the perception of surface material. Curr. Biol..

[132] Kim J, Marlow PJ, Anderson BL (2012). The dark side of gloss. Nat. Neurosci..

[133] Marlow PJ, Kim J, Anderson BL (2012). The perception and misperception of specular surface reflectance. Curr. Biol..

[134] Fleming RW, Nishida S, Gegenfurtner KR (2015). Perception of material properties. Vis. Res..

[135] Wurm MF, Ariani G, Greenlee MW, Lingnau A (2016). Decoding concrete and abstract action representations during explicit and implicit conceptual processing. Cereb. Cortex.

[136] Kriegeskorte N, Simmons WK, Bellgowan PS, Baker CI (2009). Circular analysis in systems neuroscience: The dangers of double dipping. Nat. Neurosci..

[137] Moeller S (2010). Multiband multislice GE-EPI at 7 tesla, with 16-fold acceleration using partial parallel imaging with application to high spatial and temporal whole-brain fMRI. Magn. Reson. Med..

[138] Lacadie CM, Fulbright RK, Rajeevan N, Constable RT, Papademetris X (2008). More accurate Talairach coordinates for neuroimaging using non-linear registration. Neuroimage.

[139] Lakens D (2013). Calculating and reporting effect sizes to facilitate cumulative science: A practical primer for t-tests and ANOVAs. Front. Psychol..

[140] Olejnik S, Algina J (2003). Generalized eta and omega squared statistics: Measures of effect size for some common research designs. Psychol. Methods.

[141] Bakeman R (2005). Recommended effect size statistics for repeated measures designs. Behav. Res. Methods.

