# Supplementary material for: Human brain activity reflecting facial attractiveness from skin reflection
Source: Sci Rep. 2021 Feb 22;11:3412. doi: 10.1038/s41598-021-82601-w (PMC7900112; doi:10.1038/s41598-021-82601-w)
Supplement: Supplementary file 1 — Supplementary Information. [file 41598_2021_82601_MOESM1_ESM.doc]

**Supplementary information**

**"Human brain activity reflecting facial attractiveness from skin reflection"**

Yuichi Sakano, Atsushi Wada, Hanako Ikeda, Yuriko Saheki, Keiko Tagai, Hiroshi Ando


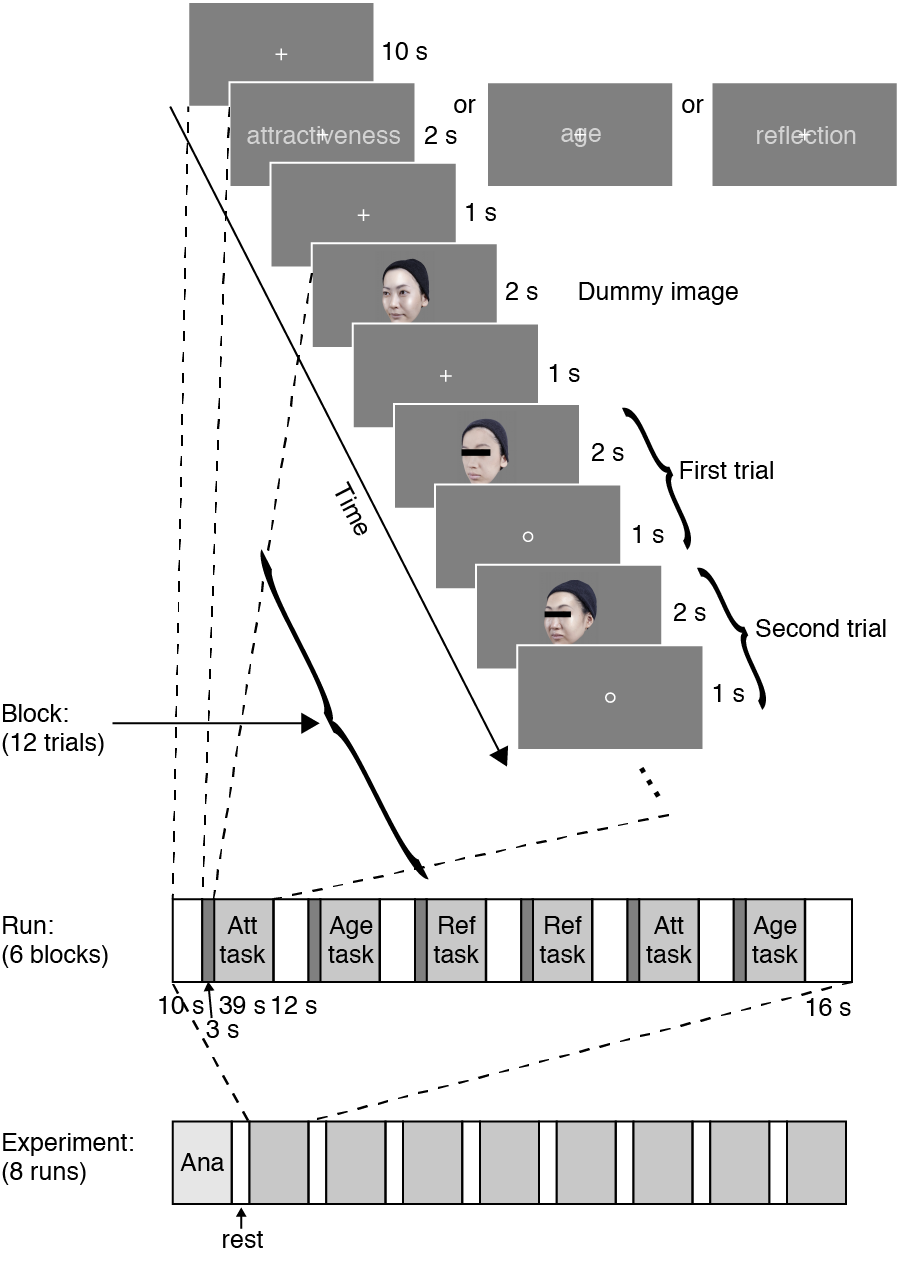


**Figure S1.** Time course of the fMRI experiment. The eyes of some models are occluded in this figure to protect personal data but were presented in the experiment. Att, Attractiveness; Ref, Skin Reflection; Ana, Anatomical scan.


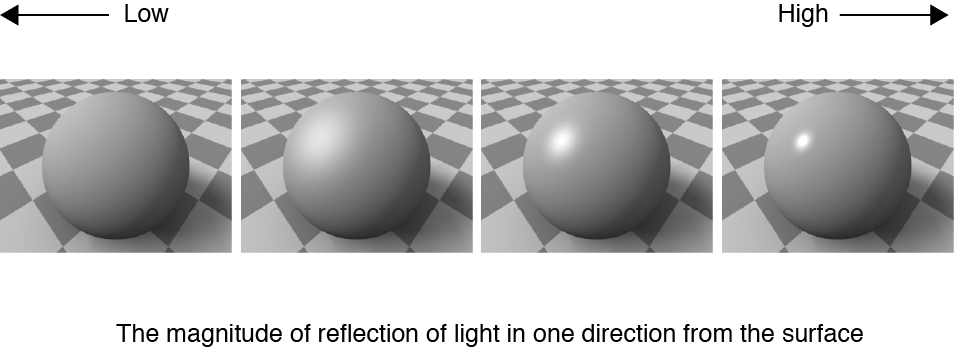


**Figure S2.** Figure used in the instructions to explain the definition of the magnitude of reflection. Images were rendered with CG software (Autodesk 3ds Max 2010). In every image, a sphere on a checkerboard was illuminated from both a parallel light from left and above with the intensity of 179/255 and a uniform light field from all directions with the intensity of 128/255. The sphere was rendered with the Phong model^S1^ with zero ambient reflectance and the diffuse reflectance of 150/255. The specular reflectance and specular directivity were 200/255 × 0.0 and 0 (i.e. completely matte) for the leftmost image, 200/255 × 0.4 and 20 for the second image from the left, 200/255 × 0.6 and 40 for the second image from the right, and 200/255 × 0.8 and 60 for the rightmost image.


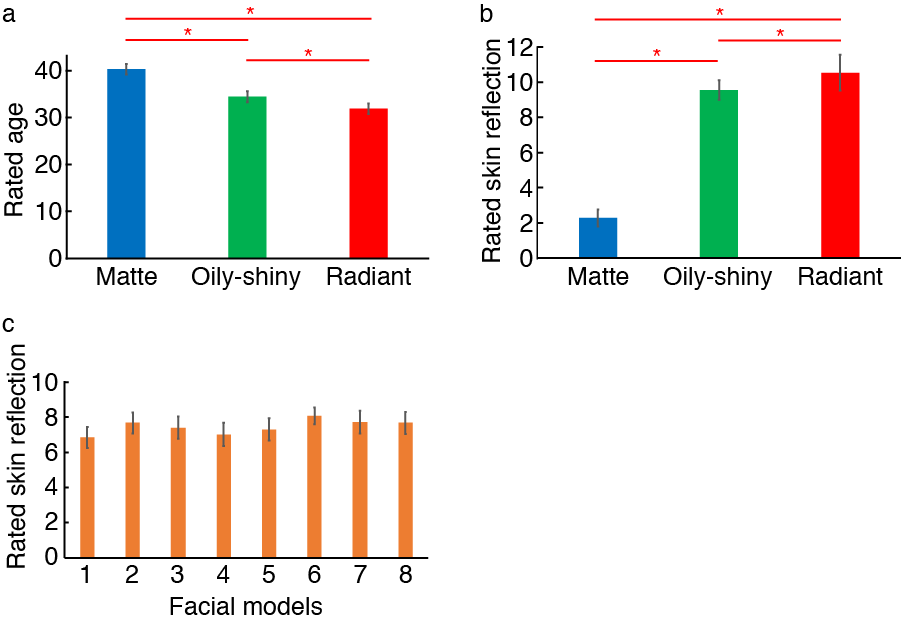


**Figure S3.** Rated age (a) and skin reflection (b), (c). (a) Rated age was highest in matte, followed by oily-shiny, and radiant skins (two-way repeated-measures ANOVA with independent variables of skin reflection and facial model, *F*(2,30) = 191.89, all *p* < 0.0001; Tukey's HSD post-hoc test for skin reflection, all *p* < 0.05). Values of the effect size measures *η*_G_^2^ and *ω*_G_^2^ of all skin reflection types were 0.313 and 0.296, respectively^S2,S3^. Hedges' *g_av_* was 1.216, 0.528, and 1.790, for oily-shiny vs. matte, radiant vs. oily-shiny, and radiant vs. matte, respectively^S4,S5^. (b) Rated skin reflection was highest for radiant skin, followed by oily-shiny skin and matte skin (two-way repeated-measures ANOVA with independent variables of skin reflection and facial model, *F*(2,30) = 559.20, *p* < 0.0001 for skin reflection; Tukey's HSD post-hoc test, all *p* < 0.05 for skin reflection). Values of *η*_G_^2^ and *ω*_G_^2^ of all skin reflection types were 0.595 and 0.578, respectively. Hedges' *g_av_* was 3.330, 0.285, and 2.461, for oily-shiny vs. matte, radiant vs. oily-shiny, and radiant vs. matte, respectively. (c) In rated skin reflection, we did not find a statistically significant main effect of the facial model or interaction in the same ANOVA (*F*(7,105) = 1.65, *p* = 0.121 for facial model, *p* = 0.093 for interaction), implying that the skin reflection was well-equalized across the different facial models used for generating the facial images. Error bars indicate ±1 SEM across subjects. **p* < 0.05.


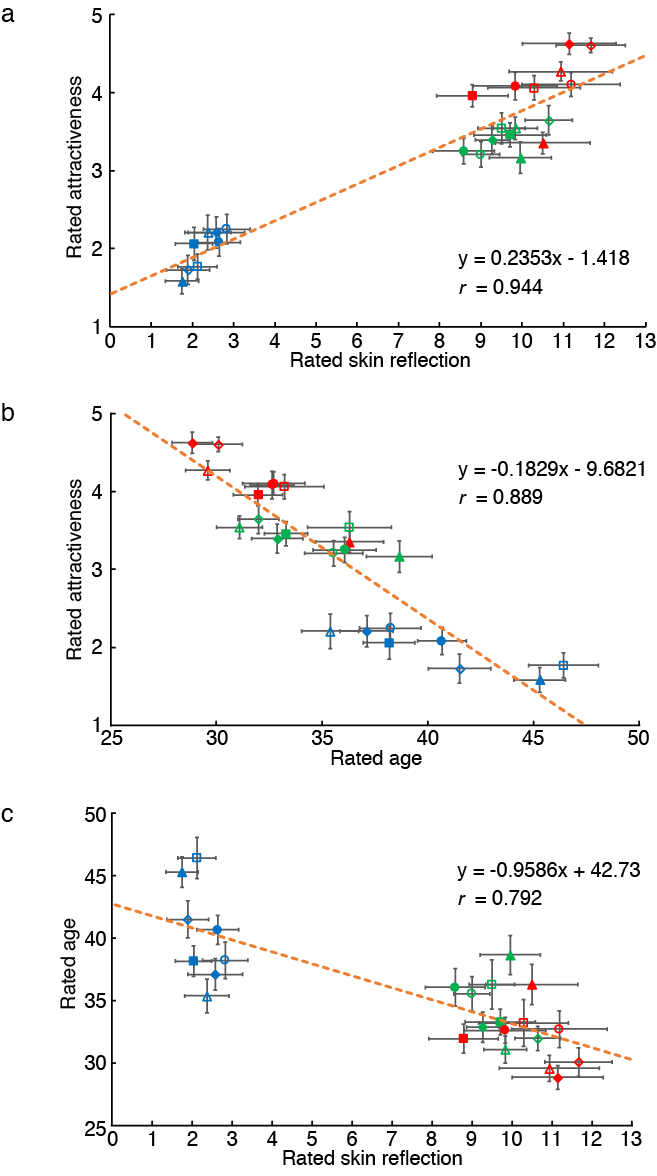


**Figure S4.** Relationships between rated skin reflection and rated attractiveness (a),

rated age and rated attractiveness (b), and rated skin reflection and rated age (c).

Error bars indicate ±1 SEM across subjects.


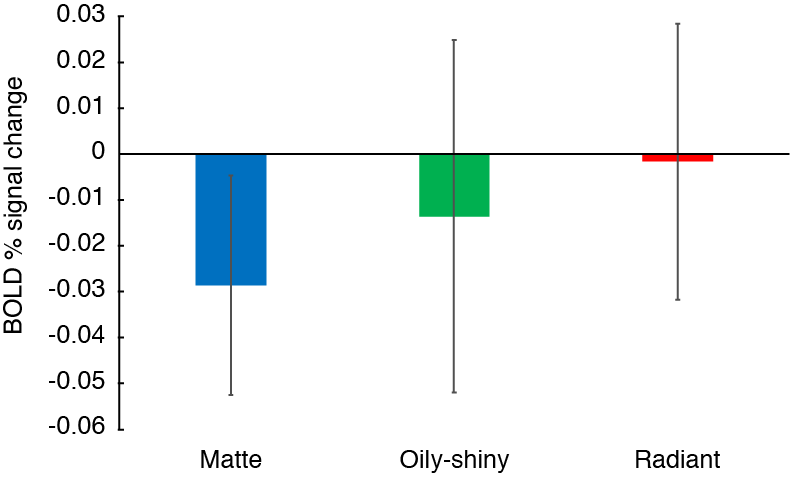


**Figure S5.** Averaged activation in the medial part of the orbitofrontal cortex (mOFC) whilst observing faces with matte, oily-shiny, and radiant skin. Error bars indicate ±1 SEM across subjects.


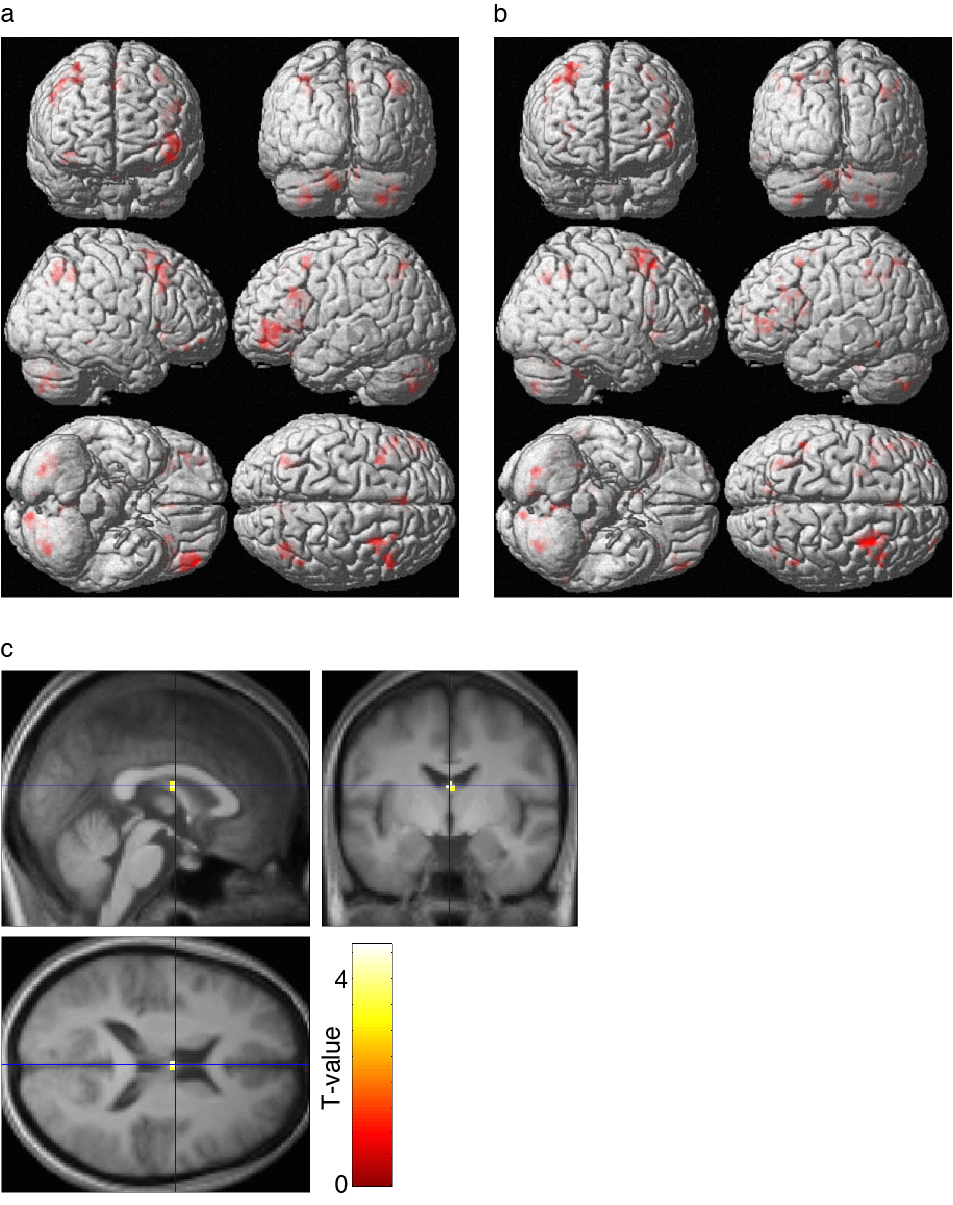


**Figure S6.** Results of comparisons of brain activation between different tasks other than the contrast of Att > Ref. Age > Ref (a), Age > Att (b), and Ref > Att (c). No regions were identified for the contrast of Ref > Age or Att > Age. Att, Attractiveness; Ref, Skin Reflection.

| Task | Factor | F-value | p-value |
| --- | --- | --- | --- |
| Attractiveness | Skin reflection | *F*(2,30) = 568.46 | < 0.0001 ** |
|  | Facial model | *F*(7,105) = 8.54 | < 0.0001 ** |
|  | Interaction | *F*(14,210) = 3.42 | < 0.0001 ** |
| Age | Skin reflection | *F*(2,30) = 191.89 | < 0.0001 ** |
|  | Facial model | *F*(7,105) = 28.81 | < 0.0001 ** |
|  | Interaction | *F*(14,210) = 3.33 | < 0.0001 ** |
| Skin reflection | Skin reflection | *F*(2,30) = 559.20 | < 0.0001 ** |
|  | Facial model | *F*(7,105) = 1.65 | 0.121 |
|  | Interaction | *F*(14,210) = 1.55 | 0.093 |

**Table S1.** Results of two-way repeated-measures ANOVA with independent variables of skin reflection and facial model for rating data on attractiveness, age, and skin reflection in the psychological experiments. ***p* < 0.0001.

| Region | R/L | BA | MNI coordinate | | | Cluster  size | t-value |
| --- | --- | --- | --- | --- | --- | --- | --- |
|  |  |  | x | y | z |  |  |
| Frontal cortex | | | | | | | |
| Inferior frontal gyrus, pars triangularis | L | 45 | −50 | 36 | 0 | 1004 | 6.47 |
| Inferior frontal gyrus, pars triangularis | R | 9 | 42 | 28 | 26 | 23 | 3.82 |
| Inferior frontal gyrus, pars orbitalis | L | 47 | −28 | 28 | −8 | 78 | 4.84 |
| Inferior frontal gyrus, pars orbitalis | L | 47 | −26 | 24 | −22 | 35 | 4.52 |
| Inferior frontal gyrus, pars orbitalis | R | 47 | 42 | 38 | −14 | 49 | 4.63 |
| Inferior frontal gyrus, pars orbitalis | R | 47 | 32 | 28 | −18 | 22 | 4.60 |
| Middle frontal gyrus, orbital part | L | 10 | −28 | 50 | −2 | 18 | 4.48 |
| Middle frontal gyrus, orbital part | R | 10 | 34 | 50 | −10 | 37 | 4.81 |
| Middle frontal gyrus | L | 6 | −36 | 12 | 52 | 105 | 5.59 |
| Middle frontal gyrus | R | N/A | 44 | 20 | 50 | 455 | 5.24 |
| Medial frontal gyrus | L | 8 | −2 | 30 | 48 | 125 | 6.23 |
| Gyrus rectus | L | N/A | 0 | 28 | −28 | 19 | 4.60 |
| Supplementary motor area | L | 6 | 0 | 20 | 62 | 22 | 4.16 |
| Parietal cortex | | | | | | | |
| Inferior parietal lobule | L | 7 | −30 | −56 | 36 | 249 | 4.99 |
| Angular gyrus | R | 39 | 36 | −64 | 44 | 336 | 4.72 |
| Precuneus | R | 31 | 4 | −58 | 36 | 118 | 4.23 |
| Temporal cortex | | | | | | | |
| Middle temporal gyrus | R | 21 | 56 | −38 | −10 | 16 | 4.10 |
| Insula | | | | | | | |
| Insula | R | N/A | 30 | 22 | 2 | 40 | 4.11 |
| Insula | R | 44 | 46 | 18 | 2 | 24 | 4.22 |
| Insula | R | 45 | 38 | 28 | 0 | 23 | 4.09 |
| Basal ganglia | | | | | | | |
| Caudate nucleus | R | 48 | 10 | 6 | 12 | 19 | 4.45 |
| Putamen | R | N/A | 26 | 18 | −8 | 18 | 4.64 |
| Globus pallidus | L | 51 | −16 | 2 | 2 | 24 | 4.50 |
| Thalamus | | | | | | | |
| Thalamus | L | N/A | −2 | −32 | −2 | 44 | 5.00 |
| **Continued** | | | | | | | |
| Thalamus | L | N/A | −12 | −20 | −10 | 17 | 5.24 |
| Thalamus | R | 50 | 12 | −10 | 2 | 37 | 4.99 |
| Cerebellum | | | | | | | |
| Crus I of cerebellar hemisphere | R | N/A | 30 | −70 | −28 | 69 | 4.10 |
| Crus I of cerebellar hemisphere | R | N/A | 10 | −80 | −24 | 36 | 4.34 |
| Crus II of cerebellar hemisphere | L | N/A | −34 | −70 | −38 | 423 | 5.34 |
| Crus II of cerebellar hemisphere | R | N/A | 28 | −78 | −48 | 280 | 5.19 |
| Lobule III of cerebellar hemisphere | R | N/A | 10 | −26 | −24 | 16 | 4.20 |
| Lobule X of vermis |  | N/A | 4 | −52 | −26 | 136 | 5.45 |

**Table S2.** Brain regions showing significant activation in the contrast of Age task – Reflection task. This contrast exhibited significant activity in the inferior parietal lobule (IPL) and middle temporal gyri, which have been reported as regions involved in the processing of facial age from unspecified cues^S6^. However, this contrast also showed activity in many other regions that have been reported as being involved in orienting attention and executive control of attention, including the middle and inferior frontal gyri, the IPL (overlapping with the previously reported age-processing regions), the precuneus, and the thalamus^S7-S9^. Thus, we discontinued further analysis of brain activity related to processing of facial age, and focused on facial attractiveness. The region of the peak voxel was identified by using the automated anatomical labeling (AAL) atlas^S10^. The Brodmann area was determined by using Yale BioImage Suite Package^S11^ (http://sprout022.sprout.yale.edu/mni2tal/mni2tal.html). BA, Brodmann area; L, left hemisphere; R, right hemisphere.

| Region | R/L | BA | MNI coordinate | | | Cluster  size | t-value |
| --- | --- | --- | --- | --- | --- | --- | --- |
|  |  |  | x | y | z |  |  |
| Frontal cortex | | | | | | | |
| Inferior frontal gyrus, pars triangularis | L | 44 | −36 | 22 | 16 | 281 | 5.02 |
| Inferior frontal gyrus, pars triangularis | L | 45 | −42 | 34 | 4 | 149 | 5.33 |
| Inferior frontal gyrus, pars triangularis | L | 45 | −40 | 20 | 6 | 85 | 4.55 |
| Inferior frontal gyrus, pars triangularis | L | 46 | −50 | 36 | 14 | 24 | 3.99 |
| Inferior frontal gyrus, pars opercularis | R | 44 | 48 | 18 | 14 | 43 | 4.11 |
| Middle frontal gyrus | L | 6 | −36 | 12 | 52 | 60 | 6.03 |
| Middle frontal gyrus | L | 10 | −30 | 60 | 12 | 48 | 3.85 |
| Middle frontal gyrus | R | 6 | 32 | 10 | 56 | 557 | 5.45 |
| Middle frontal gyrus | R | N/A | 36 | 62 | 6 | 24 | 4.01 |
| Medial frontal gyrus | L | 8 | 0 | 32 | 48 | 65 | 4.44 |
| Superior frontal gyrus | L | 6 | −24 | 0 | 62 | 34 | 4.09 |
| Parietal cortex | | | | | | | |
| Superior parietal lobule | L | 7 | −16 | −72 | 54 | 47 | 4.01 |
| Precuneus | R | 7 | 8 | −70 | 50 | 60 | 4.19 |
| Precuneus | R | N/A | 22 | −52 | 26 | 31 | 4.11 |
| Inferior parietal lobule | L | 7 | −32 | −60 | 42 | 341 | 4.81 |
| Inferior parietal lobule | R | 39 | 48 | −56 | 46 | 80 | 4.28 |
| Angular gyrus | R | 39 | 38 | −68 | 40 | 108 | 4.82 |
| Temporal cortex | | | | | | | |
| Middle temporal gyrus | R | 21 | 54 | −42 | −10 | 50 | 4.21 |
| Inferior temporal gyrus | L | 37 | −54 | −50 | −12 | 27 | 4.31 |
| Insula | | | | | | | |
| Insula | R | 13 | 28 | 20 | −8 | 96 | 4.64 |
| Insula | R | 13 | 34 | 20 | 8 | 43 | 4.20 |
| Cingulate cortex | | | | | | | |
| Midcingulate area | R | 8 | 6 | 28 | 34 | 20 | 4.17 |
| Basal ganglia | | | | | | | |
| Caudate nucleus | R | 48 | 12 | 8 | 10 | 155 | 5.78 |
| Thalamus | | | | | | | |
| **Continued** | | | | | | | |
| Thalamus | L | 50 | −14 | −26 | 10 | 30 | 4.24 |
| Thalamus | L | N/A | −22 | −30 | 2 | 19 | 4.75 |
| Thalamus | R | 50 | 4 | −10 | 2 | 25 | 5.66 |
| Cerebellum | | | | | | | |
| Crus I of cerebellar hemisphere | R | N/A | 24 | −66 | −36 | 48 | 4.70 |
| Crus I of cerebellar hemisphere | R | N/A | 44 | −58 | −28 | 34 | 4.48 |
| Crus II of cerebellar hemisphere | L | N/A | −34 | −72 | −48 | 117 | 5.52 |
| Crus II of cerebellar hemisphere | R | N/A | 26 | −76 | −46 | 142 | 5.27 |
| Lobule III of cerebellar hemisphere | L | N/A | −8 | −30 | −26 | 18 | 5.25 |
| Lobule III of cerebellar hemisphere | R | N/A | 14 | −36 | −28 | 44 | 4.63 |
| Lobule IV, V of cerebellar hemisphere | L | N/A | −28 | −36 | −34 | 24 | 4.31 |
| Lobule IV, V of cerebellar hemisphere | L | N/A | −18 | −32 | −26 | 16 | 4.49 |
| Lobule VI of cerebellar hemisphere | R | N/A | 10 | −80 | −20 | 81 | 4.70 |
| Lobule VI of cerebellar hemisphere | R | N/A | 32 | −42 | −36 | 47 | 4.85 |
| Lobule VIII of vermis |  | N/A | −2 | −60 | −36 | 280 | 4.77 |

**Table S3.** Brain regions showing significant activation in the contrast of Age task – Attractiveness task. BA, Brodmann area; L, left hemisphere; R, right hemisphere.

| Region | R/L | BA | MNI coordinate | | | Cluster  size | t-value |
| --- | --- | --- | --- | --- | --- | --- | --- |
|  |  |  | x | y | z |  |  |
| Thalamus | L | N/A | 0 | −6 | 16 | 17 | 4.65 |

**Table S4.** Brain regions showing significant activation in the contrast of Reflection task – Attractiveness task. BA, Brodmann area; L, left hemisphere; R, right hemisphere.

**Supplementary Methods: generating stimuli**

The stimuli used in the present study were a subset of those used in our previous study that demonstrated the effects of skin reflection on facial attractiveness^S12^. The previous study used not only the images used in the present study, but also facial images with reflection only on cheeks or T-zones. Among all those conditions, the three conditions used in the present study showed the most prominent effects of the types of reflection on facial attractiveness. That is, attractiveness prominently increased from faces with matte, to oily-shiny, to radiant skin. Accordingly, we used those stimuli of the three conditions in the present study.

**1. Procedure for generating the stimulus images**

The radiant, oily-shiny, and matte skins are skin reflection types determined by visual impression rather than by physical reflectance properties^S13,S14^. In the present study, we generated images for each type of skin reflection for each of nine female models. To maximize the impression of the skin reflection types, and to minimize the unnaturalness of the generated facial images, the procedure for generating the stimulus images involved a photo-taking phase and a retouching phase, utilizing three techniques. (1) We applied several types of cosmetics so that the skin composition exhibited the physical characteristics of reflection inherent in the three skin reflection types (i.e., radiant, oily-shiny, or matte)^S15,S16^. (2) We took advantage of the effects of 3D shape on the perception of reflection^S17-S19^. Specifically, we generated the facial images with radiant skin by combining highlights on the convex cheeks of the smiling faces with the faces of neutral expression (without the appearance of having been retouched). (3) To control the degree of texture fineness, a hallmark of the skin reflection types^S14,S15^, we used cosmetics, as mentioned above, as well as manipulated the lighting conditions and retouched the facial photos. Thus, the facial photos were retouched because the combination of cosmetics and the lighting control was insufficient to control the skin reflection types.

**2. The photo-taking phase**

**2.1. Taking into consideration previous reports discussing the expression of different types of skin reflection**

To express the three types of skin reflection on each face, we manipulated lighting conditions and used several types of cosmetics. To choose lighting conditions and cosmetics, we considered the results of previous studies^S14,S16,S20^. These studies reported that different combinations of the intensities of specular and diffuse reflection can give different impressions, such as radiant or oily-shiny skin. Masuda and colleagues (2017)^S14^ reported that the impression of oily-shiny skin was dominant when the specular and diffuse reflection were intense and weak, respectively, while the impression of radiant skin emerged when both types of reflection were intense.

Specular reflection images cause unique skin appearance, not only because of the sebum distributed on the skin surface but also because of the surface roughness^S16^. In addition, it was reported that texture roughness (i.e., distance between skin surface furrows) is a hallmark of skin which exhibits intense specular reflection and weak diffuse reflection, which corresponds to an oily-shiny skin^S14^. On the other hand, Ojima and colleagues (1993)^S21^ reported that unlike specular reflection, skin roughness was not visible on diffuse reflection images, including tiny wrinkles and pores. Similarly, skin that exhibits a large amount of diffuse reflection has a fine texture and holds plenty of moisture^S15^.

**2.2. General methods**

To generate different types of facial image, we artificially expressed the characteristics of the diffuse and specular reflection on the skin. To express the characteristics of diffuse reflection (fine texture and holding plenty of moisture), it was necessary to generate images of smooth skin. To do so, when taking photos, we used diffuse illumination, which produced smooth looking skin, along with cosmetic products that made facial skin smooth.

To generate facial images with radiant skin, in addition to such expression of diffuse reflection, we expressed specular highlights by using bright diffuse illumination.

To generate facial images with oily-shiny skin, we used cosmetic products in which there was plenty of oil in the base material along with non-diffuse illumination. This was because previous studies have reported that oily-shiny skins hold plenty of sebum^S22,S23^, and that people with faster production of sebum are more conscious of facial oiliness^S24^.

The facial expression was neutral or smiling. Highly convex cheeks and other parts of the smiling face were used as strong specular highlights when generating the facial images with radiant and oily-shiny skin in the subsequent retouching phase.

The models sat on a chair with their whole body and face directed to the right at a 45 degree angle from the camera. As a result, the left side of the face was directed towards the camera (a digital single-lens reflex camera, Canon, Japan). A chin rest was used to control the facial direction.

**2.3. Illumination conditions**

Photos of all models were taken under diffuse and non-diffuse illumination conditions. Under the non-diffuse illumination condition, we used one side-light (Comet strobe light and strobe head) and two strobe lights fitted with umbrella-shaped reflectors. All three lamps were used at an output of 200 W. In the diffuse illumination condition, one side-light and one strobe light equipped with a diffuser were used at an output of 112 W.

**2.4. Skin conditions by using cosmetics**

Before taking photos, all the models washed their faces, and then performed skincare with lotion and emulsion to avoid dry skin. They then rested for around 10 minutes until the skin redness subsided. Makeup was applied by a professional makeup artist to achieve the different styles specified. After the artist drew on the models' eyebrows, photos were taken with three patterns of applied cosmetics: (1) applying solid foundation only to the whole face, (2) applying cosmetic oil to the whole face to prevent dry skin after applying the foundation, and (3) applying cosmetic moisturizing gel to the cheeks only and cosmetic oil to the whole face, both after applying foundation to the whole face.

We took preliminary photos to confirm that the foundation achieved a fine skin texture. Therefore, the major portions of the faces only with the foundation in the photos were clipped and used as (1) the image portions expressing diffuse reflection to generate the images of faces with radiant skin and (2) the base face images.

During the preliminary photo taking, it was also confirmed that photos of the faces coated with cosmetic oil and gel expressed oily-shiny reflection as well as skin roughness. Thus, some portions of these photos were clipped and used as oily-shiny highlights to generate the images of the faces with oily-shiny skin.

**3. The retouching phase**

Facial photos were retouched using CG software (Adobe Photoshop CC 2017). The gamma value of an LCD display (Eizo, Japan) used for retouching was 2.2. Each face was clipped from the photo and retouched on an image with a resolution of 2995 (W) × 4493 (H) pixels. We first generated a base face image of each model. Then, we transplanted convex facial portions from photographs taken variously onto the base face of the same model to generate three types of image: faces with radiant, oily-shiny, and matte skins. The convex facial portions included the forehead, nose ridge, cheeks, and chin. Areas of these portions were manipulated so that they appeared natural according to the facial shape of each model. The composite images were shrunk to 719 (W) × 1078 (H) pixels for presentation in the experiments. A person in charge of image retouching and five authors as well as Kei Fukazawa, Tomomi Yagasaki, and Ayaka Ichimi at the cosmetic marketing and development division of Shiseido Company, Limited confirmed that the areas, shapes, and intensities appeared natural (with the appearance of not having been retouched).

**3.1. Methods to generate base faces**

We generated a base face of each model by removing strong highlights in the convex facial portions from the photo of the same model's face with neutral expression and only foundation applied, taken under diffuse illumination. To remove the highlights, we transplanted the non-highlighted portion of the skin onto the highlights. Moderate or weak highlights remained untouched. Skin eruptions, spots, and scratches were also removed from the original face.

**3.2. Methods to generate faces with radiant skin**

An image of each model's face with radiant skin was generated by combining the base face with two types of image. The first was highlights with the characteristics of diffuse reflection (fine texture and holding plenty of moisture), and the second was strong specular highlights that gave an impression of smooth skin. Specifically, the highlights with the characteristics of diffuse reflection were clipped from the photo of the same model's face with neutral expression and with only foundation applied, taken under diffuse illumination. Similarly, strong specular highlights were clipped from the photo of the same model's neutral and the smiling face with only foundation applied, taken under non-diffuse illumination. The parts of the smiling faces that we used depended on the model we used to achieve the impression of faces with radiant skin, because there were considerable differences between the models in skin reflection type.

**3.3. Methods to generate oily-shiny faces**

An image of each model's face with oily-shiny skin was generated by combining the base face with specular highlights expressing oily-shiny reflection as well as skin roughness. Specifically, into the base face photo, we transplanted portions of specular highlights of the model's neutral and smiling face coated with oil and foundation, or with oil, gel, and foundation, taken under non-diffuse illumination.

**3.4. Methods to generate matte faces**

To generate an image of each model's matte face, we removed all highlights, including moderate or weak ones, from the base face.

**Supplementary References**

1. Phong, B. T. Illumination for computer generated pictures. *Commun. ACM* **18**, 311-317, (1975).
2. Olejnik, S. & Algina, J. Generalized eta and omega squared statistics: measures of effect size for some common research designs. *Psychol. Methods* **8**, 434-447, doi:10.1037/1082-989X.8.4.434 (2003).
3. Bakeman, R. Recommended effect size statistics for repeated measures designs. *Behav. Res. Methods* **37**, 379-384, doi:10.3758/bf03192707 (2005).
4. Cumming, G. *Understanding the new statistics: effect sizes, confidence intervals, and meta-analysis*. (Routledge, 2012).
5. Lakens, D. Calculating and reporting effect sizes to facilitate cumulative science: a practical primer for t-tests and ANOVAs. *Front. Psychol.* **4**:863, 1-12, doi:10.3389/fpsyg.2013.00863 (2013).
6. Homola, G. A., Jbabdi, S., Beckmann, C. F. & Bartsch, A. J. A brain network processing the age of faces. *PLoS One* **7**, e49451, doi:10.1371/journal.pone.0049451 (2012).
7. Liu, T., Slotnick, S. D., Serences, J. T. & Yantis, S. Cortical mechanisms of feature-based attentional control. *Cereb. Cortex* **13**, 1334-1343 (2003).
8. Yantis, S. & Serences, J. T. Cortical mechanisms of space-based and object-based attentional control. *Curr. Opin. Neurobiol.* **13**, 187-193 (2003).
9. Petersen, S. E. & Posner, M. I. The attention system of the human brain: 20 years after. *Annu. Rev. Neurosci.* **35**, 73-89, doi:10.1146/annurev-neuro-062111-150525 (2012).
10. Tzourio-Mazoyer, N. et al. Automated anatomical labeling of activations in SPM using a macroscopic anatomical parcellation of the MNI MRI single-subject brain. *Neuroimage* **15**, 273-289, doi:10.1006/nimg.2001.0978 (2002).
11. Lacadie, C. M., Fulbright, R. K., Rajeevan, N., Constable, R. T. & Papademetris, X. More accurate Talairach coordinates for neuroimaging using non-linear registration. *Neuroimage* **42**, 717-725, doi:10.1016/j.neuroimage.2008.04.240 (2008).
12. Ikeda *et al*. Facial radiance influences facial attractiveness and affective impressions of faces. *Int. J. Cosmet. Sci.* (accepted).
13. Petitjean, A. *et al.* Skin radiance: how to quantify? Validation of an optical method. *Skin Res. Technol.* **13**, 2-8, doi:10.1111/j.1600-0846.2006.00174.x (2007).
14. Masuda, Y., Yagi, E., Oguri, M. & Kuwahara, T. Development of a quantitative method for evaluation of skin radiance and its relationship with skin surface topography. *J. Soc. Cosmet. Chem. Japan* **51**, 211-218, doi:10.5107/sccj.51.211 (2017) [in Japanese].
15. Masuda, Y., Kunizawa, N. & Takahashi, M. Methodology for evaluation of skin transparency and the efficacy of an essence that can improve skin transparency. *J. Soc. Cosmet. Chem. Japan* **39**, 201-208, doi:10.5107/sccj.39.3_201 (2005) [in Japanese].
16. Fujii, M., Misaki, Y. & Sasaki, I. Application of image processing technique for facial gloss evaluation. *J. Soc. Cosmet. Chem. Japan* **43**, 72-78, doi:10.5107/sccj.43.72 (2009) [in Japanese].
17. Nishida, S. & Shinya, M. Use of image-based information in judgments of surface-reflectance properties. *J. Opt. Soc. Am. A Opt. Image Sci. Vis.* **15**, 2951-2965 (1998).
18. Wijntjes, M. W. & Pont, S. C. Illusory gloss on Lambertian surfaces. *J. Vis.* **10**, 13, 11-12, doi:10.9.13 [pii] 10.1167/10.9.13 (2010).
19. Marlow, P. J., Kim, J. & Anderson, B. L. The perception and misperception of specular surface reflectance. *Curr. Biol.* **22**, 1909-1913, doi:10.1016/j.cub.2012.08.009 (2012).
20. Ohtsuki, R., Hikima, R., Sakamaki, T. & Tominaga, S. Evaluation method of make-up deterioration using a multi-band facial image. *Journal of the Color Science Association of Japan* **37**, 200-201 (2013) [in Japanese].
21. Ojima, N., Haneishi, H. & Miyake, Y. The appearance of skin with make-up (II): Analysis on surface topography of skin with make-up. *Bull. Soc. Sci. Photogr. Japan* **56**, 264-269, doi:10.11454/photogrst1964.56.264 (1993) [in Japanese].
22. Mizukoshi, K. & Akamatsu, H. The investigation of the skin characteristics of males focusing on gender differences, skin perception, and skin care habits. *Skin Res. Technol.* **19**, 91-99, doi:10.1111/srt.12012 (2013).
23. Nouveau-Richard, S. et al. Oily skin: specific features in Chinese women. *Skin Res. Technol.* **13**, 43-48, doi:10.1111/j.1600-0846.2006.00185.x (2007).
24. Torizuka, M., Nagatani, N., Syoji, T., Asahi, M. & Takano, S. Study of various factors which cause the wear-off during the foundation application. *J. Soc. Cosmet. Chem. Japan* **28**, 350-358, doi:10.5107/sccj.28.350 (1995) [in Japanese].
